# Histotripsy treatment reduces tumor burden and extends survival in an orthotopic mouse model of osteosarcoma

**DOI:** 10.3389/fonc.2026.1807753

**Published:** 2026-04-13

**Authors:** Elliana R. Vickers, Alayna N. Hay, Victor A. Lopez, Lauren N. Ruger, Ny T. C. Luong, Julianna M. Diodato, Triniti Antony Baskar, Alyssa Sze, Maosen Wang, Steven B. Soliman, Gunjan B. Malhotra, Sheryl Coutermarsh-Ott, John H. Rossmeisl, Eli Vlaisavljevich, Joanne Tuohy

**Affiliations:** 1 Virginia Tech Animal Cancer Care and Research Center, Virginia-Maryland College of Veterinary Medicine, Roanoke, VA, United States; 2 Department of Biomedical Engineering and Mechanics, Virginia Polytechnic Institute and State University, Blacksburg, VA, United States; 3 School of Neuroscience, Virginia Polytechnic Institute and State University, Blacksburg, VA, United States; 4 Department of Biological Sciences, Virginia Polytechnic Institute and State University, Blacksburg, VA, United States; 5 Virginia Tech Carillion School of Medicine, Virginia Polytechnic Institute and State University, Roanoke, VA, United States; 6 Fralin Biomedical Research Institute, Virginia Polytechnic Institute and State University, Roanoke, VA, United States; 7 Division of Musculoskeletal Radiology, Department of Radiology, University of Michigan/Michigan Medicine, Ann Arbor, MI, United States; 8 Department of Biomedical Sciences and Pathobiology, Virginia-Maryland College of Veterinary Medicine, Blacksburg, VA, United States; 9 Department of Small Animal Clinical Sciences, Virginia-Maryland College of Veterinary Medicine, Blacksburg, VA, United States

**Keywords:** ablation, bone tumor, focused ultrasound, histotripsy, mouse, orthotopic, osteosarcoma, rodent

## Abstract

**Background:**

Osteosarcoma (OS) is a devastating primary bone tumor. Histotripsy, a non-invasive ablation modality that mechanically destroys tissue with focused ultrasound, has shown promise for the treatment of OS. The goal of this study is to characterize the ablative effects after histotripsy treatment in an orthotopic mouse model of OS.

**Methods:**

Mice (n=53) were grouped based on tumor volume on the day of histotripsy treatment: treated-small (<200 mm^3^, 143 ± 35 mm^3^) and treated-large (>200 mm^3^, 268 ± 44 mm^3^). Histotripsy outcomes were evaluated and compared on MRI and histopathology at different acute (1 and 3) and sub-acute (7 and 14) days post-treatment (DPT). 5 mice were survived up to 30 DPT, and factors correlating with survival were evaluated.

**Results:**

At 1 DPT, treated tumors show significant hemorrhage (p < 0.0001), loss of contrast enhancement (34% decrease, *p* = 0.0480), and reduction in tumor volume (treated-small: 74% decrease from untreated, *p* = 0.0002; treated-large, 47% decrease from untreated, *p* = 0.0002). There was no significant change in treated-small tumor volume from 7 to 14 DPT (*p* = 0.7302), and treatment zones showed significant involution by 14 DPT (75% decrease, *p* = 0.0055). Only treated-small mice survived beyond 14 DPT, and survival was significantly extended by histotripsy treatment (*p* < 0.0001) by a median of 15 days compared to untreated OS mice.

**Conclusions:**

Histotripsy treatment significantly reduced tumor burden and extended survival compared to untreated controls. These results highlight histotripsy’s promise as a safe and effective non-invasive treatment for OS.

## 1 Introduction

Osteosarcoma (OS) is a locally aggressive and highly metastatic primary bone tumor, occurring most commonly in children and adolescents (1–4) and also in pet dogs (5, 6). OS is the most common primary bone tumor in humans and dogs, accounting for 55% of primary bone tumors diagnosed in children in the U.S (3, 4, 7). and 80–90% of primary bone tumors diagnosed in pet dogs (8). Standard-of-care for OS includes removal of the primary tumor via limb amputation or limb salvage surgery, combined with chemotherapy to address metastatic disease (1); however, survival rates for humans and canines have not improved in decades (9). Limb salvage surgeries are associated with risks such as infection, implant failure, local recurrence, and fracture, often requiring multiple surgeries or eventual limb amputation (10, 11). Furthermore, these surgeries can impair patient mobility and limb function (12), and despite these aggressive treatment measures, prognosis remains poor. The size of OS tumors at time of diagnosis varies, as with most cancers, but typically presents at later stages and with larger tumor sizes; studies report bone sarcomas ranging from 10.7 cm to 11.3 cm (maximum diameter) (13, 14). The 5-year event-free survival rate for pediatric OS with localized disease and complete surgical remission is 64% (15), but this drops to below 30% for patients with metastases at presentation (16, 17), and an estimated 80% of OS patients have micrometastases present at diagnosis (5). A non-surgical limb-sparing treatment option for OS with the potential to improve survival outcomes is needed. Histotripsy is an emerging treatment modality which has the potential to meet this need and has recently demonstrated promise for the treatment of OS (18–21) and other solid tumors (22).

Histotripsy is the first non-invasive, non-thermal, and non-ionizing ablation modality that mechanically destroys targeted tissue with high precision via focused ultrasound (FUS) (22). The high-pressure FUS waves cause acoustic cavitation, the rapid expansion and collapse of microbubbles in tissue, effectively liquefying the targeted volume (23). The histotripsy “bubble cloud” is visible on ultrasound (US) and can be used to monitor the ablation, offering real-time image guidance during treatment. Histotripsy’s mechanism of action offers tissue selectivity and sparing of mechanically tougher critical structures, such as large blood vessels (24–26), nerves (18, 27), and healthy bone (18, 28, 29). The feasibility of histotripsy for targeting and ablating a variety of tumor and tissue types has been explored preclinically in large animal models, including porcine models for ablation of the liver (30), kidney (31), and pancreas (32–34), as well as in veterinary clinical trials investigating histotripsy for the treatment of canine brain tumors (35), feline injection site sarcomas (36), canine soft tissue sarcoma (37), and canine OS (19–21). In addition, the long-term outcomes of histotripsy has been studied in rodent models for a variety of tumor types (38), including cancers of the liver (24, 39–42), brain (43–45), kidney (27), skin (41, 42), breast (46), and pancreas (47). Histotripsy has also completed human clinical trials and received FDA approval for the treatment of liver cancer (48, 49) and is in clinical trials for ablation of tumors in the kidney [NCT05432232, NCT05820087] and the pancreas [NCT06282809].

Our team has previously investigated the safety and feasibility of using histotripsy for the treatment of OS. Our initial studies have shown safe, feasible, and effective ablation of OS in a heterotopic (subcutaneous) OS murine model (50), an orthotopic OS xenograft (immunocompromised) murine model (51), and in multiple treat-and-resect clinical trials with pet dogs (19–21). However, we have yet to explore the long-term outcomes after histotripsy ablation of OS, which is essential for the clinical advancement of histotripsy ablation for OS. To expand on this knowledge gap, the orthotopic syngeneic OS murine model can be utilized. This model has a higher degree of biological relevance than the heterotopic subcutaneous OS murine model (52, 53) and is a more feasible model for gaining foundational insights on the long-term outcomes post-histotripsy ablation than the pet dog, allowing for more comprehensive and mechanistic studies. Additionally, the immunocompetent nature of this model allows for investigations into the long-term tumor responses and survival outcomes after histotripsy in a more clinically relevant animal model of OS.

In this study, we investigate the ablative and imaging outcomes over time after histotripsy ablation in an orthotopic syngeneic OS murine model. In particular, we characterize and compare tumor tissue characteristics on both histopathology and MRI during the acute (1–3 days) and sub-acute (7–14 days) post-treatment time periods, along with a survival cohort of mice to investigate long-term outcomes. We hypothesize that 1) histotripsy will effectively ablate the targeted tumor volumes as measured by decreased viable tumor volumes on histopathology and MRI at acute timepoints; 2) histotripsy-ablated tumor volumes will resorb over time after treatment, similar to what has been observed for histotripsy ablation of liver (24, 30) and other tissues (39, 54); and 3) orthotopic OS mice treated with histotripsy will show significantly increased survival compared to untreated tumor-bearing mice. A secondary goal of this study is to evaluate treatment responses between small versus large tumors, where we hypothesize that tumors treated when small will have better outcomes.

## 2 Methods

### 2.1 Murine orthotopic OS model

The DLM8 murine OS cell line (RRID: CVCL_6669) was gifted from Dr. E Kleinman (M.D. Anderson Cancer Center, University of Texas, Houston, TX, USA) and used in a syngeneic immunocompetent C3H/HeN mouse model (Charles River, Wilmington, MA, USA. RRID: MGI:2160972). All mice were between the age of 8–10 weeks and female. DLM8 cells were maintained in Dulbecco’s modified eagle medium (DMEM) (Gibco, Thermo Fisher Scientific, Waltham, MA, USA) supplemented with 10% fetal bovine serum (Gibco) and 1% penicillin-streptomycin (Gibco). To prepare for tumor induction, cells were lifted with 1.25% trypsin and washed in 1X sterile phosphate buffered saline (PBS, Gibco). Cells were resuspended in a 50% 1X phosphate buffered saline (PBS, Gibco):Matrigel® (Corning Inc., Corning, NY, USA) solution, then 50 uL of cell suspension containing 1x10^6^ cells was injected into the right hindlimb via para-tibial injection (51, 55, 56). Mice were anesthetized briefly for tumor cell injection via inhaled isoflurane (2.0 L/min oxygen and 2% isoflurane) and a low-flow vaporizer (SomnoFlo®, Kent Scientific Corporation, Torrington, CT, USA).

**Figure 1:**
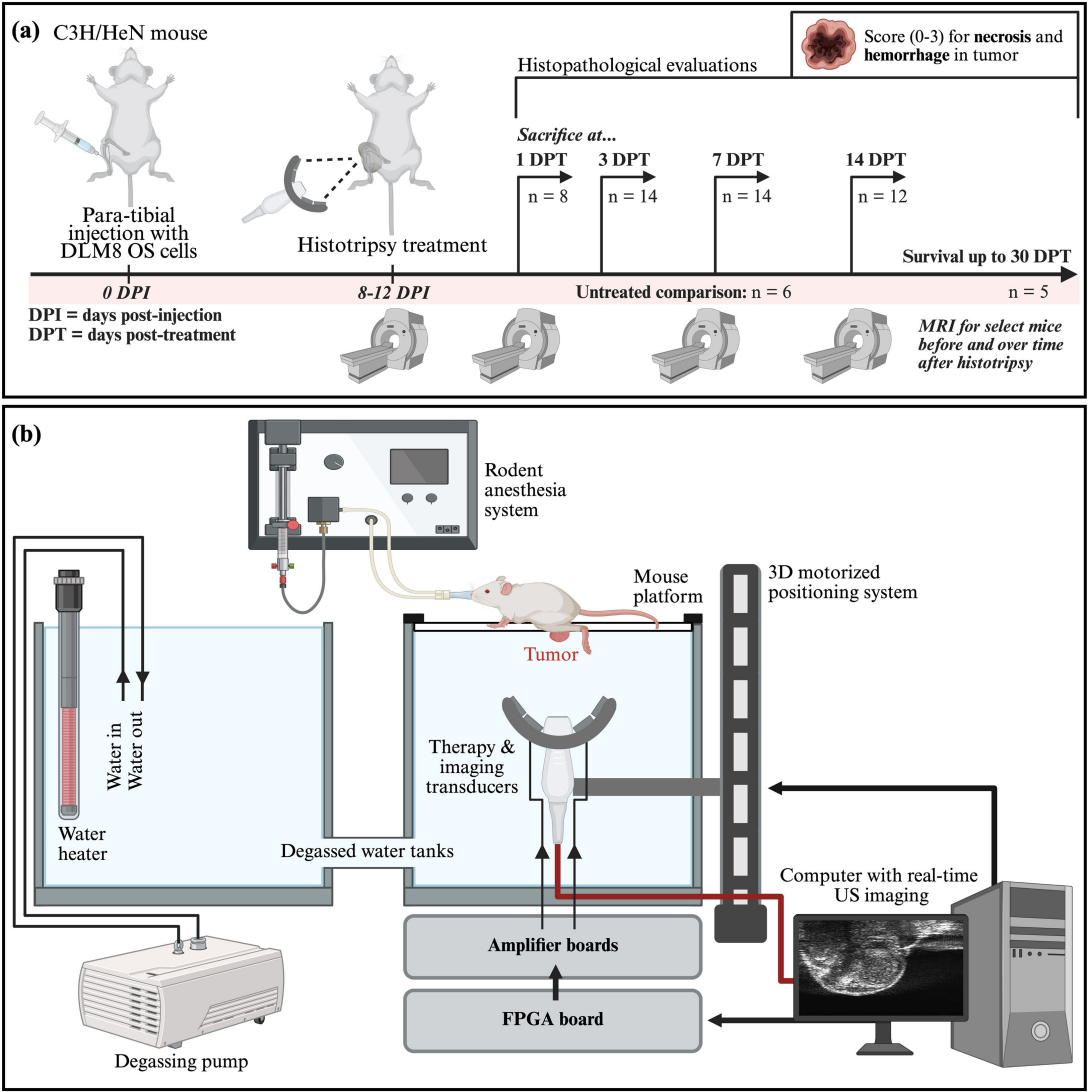
Schematic of histotripsy ablation, guided by real-time US imaging, in an orthotopic mouse model of OS. **(a)** Workflow for histotripsy study in murine orthotopic OS. **(b)** Diagram of set-up and system for histotripsy ablation of murine tumor-bearing limbs. Figure created using BioRender.com.

Histotripsy treatment was delivered between 8 to 12 days post-tumor cell injection, depending on the size of the tumor on MRI, US, and gross anatomy. Mice were grouped into small or large tumor volume treatment groups based on pre-treatment tumor volume as averaged between the three measurement modalities (<200 mm^3^ = *treated-small*, >200 mm^3^ = *treated-large*). Measured tumor volumes (from dimensional measurements with calipers or on US imaging) were calculated assuming an ellipsoidal volume ( $volume=\frac{4}{3}\times \pi \times (\frac{tumor\;length}{2})\times (\frac{tumor\;width}{2})\times (\frac{tumor\;depth}{2})$. A total of 53 mice were treated, and sacrifice was performed at the following timepoints post-histotripsy: 1 day (n = 8), 3 days (n = 14), 7 days (n = 14), and 14 days (n = 12) post-treatment (DPT). A final survival group (n = 5) was carried out for 14+ DPT until mice reached endpoint criteria (20% body weight loss or largest tumor diameter > 12 mm) or until the predetermined study endpoint of 30 DPT. An additional group of untreated mice (n = 6) followed the same tumor cell injection and monitoring protocols but did not receive histotripsy treatment and were sacrificed when they met endpoint criteria (described above). This workflow is illustrated in **Figure 1a**. The number of mice in each group was calculated based on *a priori* power analysis using previously collected data and a 90% power with a group size of 8 mice, and 100% > 10 mice was determined. All animals were used for their intended experimental group and there were no exclusion criteria. All data collected from the reported animals are presented in this manuscript. For all experiments, investigators were aware of group allocations unless otherwise noted (histopathology and MRI segmentation analysis).

Mice were monitored (weight, tumor size, and body condition) 3x weekly prior to histotripsy, as well as daily for 5 days post-treatment, and then 3-5x weekly until the point of sacrifice. If a mouse was nearing an endpoint criteria, the monitoring frequency increased to daily. Tumor size was measured over time using calipers, by obtaining at least two sets of measurements of the tumor-bearing limb (proximal to distal, cranial to caudal, and medial to lateral); the two sets of measurements at each growth monitoring timepoint were consistently obtained by two individuals (A.N.H. and E.R.V. or N.T.C.L.), and the final reported measurement is the average of the two sets of obtained measurements at each monitoring session. During tumor growth and body weight monitoring, mice were assessed for overall wellbeing by evaluating body and coat condition, along with responsiveness. Upon reaching endpoint criteria, mice were euthanized via inhaled CO_2_, with cardiac puncture as the secondary method of euthanasia. At sacrifice, a portion of the tumor was sectioned for histopathological analysis and reference to MR imaging for comparison of characteristics between MRI and histopathology. All experiments were conducted under Virginia Tech Institutional Animal Care and Use Committee approval (protocols #22-133, 23-189, and 24-021) and in accordance with the NIH Guide for the Care and Use of Laboratory Animals.

### 2.2 Histotripsy treatment

The histotripsy system and workflow used in this study has been described previously (46, 47, 50, 51) and is depicted in **Figure 1b**. Prior to histotripsy treatment, fur on the tumor-bearing limb was removed via application of a depilatory cream (Nair™, Church & Dwight Co. Inc., Ewing, NJ, USA) for 1–2 minutes. Mice were anesthetized via inhaled isoflurane (2.0 L/min oxygen and 2.5% isoflurane) for treatment and placed on a custom mouse positioning stage with their tumor-bearing limb in a tank of degassed warm (37°C) water. Histotripsy was delivered using a custom 1 MHz 8-element transducer with a coaxially aligned imaging probe with a frequency range of 10–18 MHz (L18-10L30H-4, Telemed, Vilnius, Lithuania, EU) mounted in the water below the mouse stage on a custom three-axis robotic positioning system. Imaging was performed at a frequency of 18 MHz. The positioning system and treatment planning were controlled via MATLAB (MATLAB, MathWorks, Portola Valley, CA, USA). The transducer was driven using a custom pulser designed to generate short single-cycle pulses controlled by a field-programmable gate array (FPGA) board (Altera DE0-Nano Terasic Technology, Dover, DE, USA) and powered by a high-voltage DC power supply (GENH750W, TDK-Lambda, National City, CA, USA).

Histotripsy treatment aimed to target as much of the tumor volume as possible, while avoiding cavitation on the skin surface or in the adjacent healthy muscle. Histotripsy was delivered at a pulse repetition frequency (PRF) of 500 Hz for all treatments. Treatments were applied at a dose of 500–1000 pulses per location (ppl) at a spacing of 1–1.5 mm (axial) x 0.5–0.75 mm (elevational and transverse). Across these two treatment paradigms (1000 ppl at 1.5 x 0.75 x 0.75 mm vs. 500 ppl at 1.0 x 0.5 x 0.5 mm), histopathology revealed indistinguishable ablation effects at 7 DPT, showing hemorrhage, loss of cellular architecture, and tumor cell debris; additionally, hemorrhage and necrosis, as scored on histopathology, were not significantly different at any timepoint post-treatment between the two parameter sets (*p* > 0.05 for all comparisons), and there was no significant effect of parameter set for the hemorrhage [*p* = 0.0981, F(1,39) = 2.872] or necrosis [*p* = 0.8098, F(1,39) = 0.05871] scores (**Supplementary Figure 1**).

Prior to study initiation, the histotripsy transducer was calibrated in degassed water using a fiber optic hydrophone (HFO-690, ONDA Corporation, Sunnyvale, CA, USA), and treatments were applied at peak negative pressures above the intrinsic threshold of water-based tissues (26–30 MPa) (57, 58). The pressure for each treatment was determined by increasing voltage in the center of the tumor until robust cavitation (i.e. not a few sporadic hyperechoic bubbles, but a full hyperechoic oscillating cloud of bubbles) was achieved. US imaging also showed swelling of the tumor-bearing limb throughout the treatment, and to quantify swelling on US, the tumor depth (skin to bone) was measured on same slice of the US image immediately before and after ablation. These measurements were made by two co-authors (T.A.B. and J.M.D., 1 year of experience) and averaged between the two observers.

### 2.3 Magnetic resonance imaging

MR images were acquired before histotripsy and over time after treatment for untreated, treated-small, and treated-large tumor groups, using a 9.4T Bruker® BioSpec 94/20 USR (Bruker, Billerica, MA, USA). Mice were anesthetized as described above and positioned in the MR scanner with a heated water pad for warmth (37°C) and a respiratory pad to monitor breathing. Fat-suppressed T2-weighted (T2w FS) images were acquired for all mice (echo time = 8 ms, repetition time = 2500 ms, flip angle = 180°, 0.7 mm slice thickness). Baseline MR images were acquired from n = 37 (29 to be treated and 8 untreated) tumor-bearing mice for histotripsy planning and imaging characterization. Images were additionally acquired at 1 DPT (n = 17 treated and 6 untreated), 7 DPT (n = 9 treated and 3 untreated), and 14 DPT (n = 4 treated only, as untreated mice were unable to survive to this timepoint).

For a preliminary evaluation of contrast-enhanced MRI, T1-weighted FS contrast-enhanced (T1w FS CE) images were acquired for n = 3 mice along with the T2-weighted FS images (echo time = 1 ms, repetition time = 250 ms, flip angle = 30°, 0.7 mm slice thickness). A gadolinium-based contrast agent (ProHance^®^ from BRACCO Diagnostics, Monroe, NJ, USA) was injected subcutaneously between the shoulder blades at a dose of 50 μL for a 20 g mouse, and images were acquired 5 minutes post-injection. This timing was based on contrast optimization images, where contrast was injected and images were acquired immediately post-injection and up to 30 minutes post-injection (**Supplementary Figure 2**).

MRIs were evaluated qualitatively by the primary author (E.R.V., 3 years of experience) as well as two board-certified musculoskeletal radiologists (S.B.S. and G.B.M., 15 and 3 years of experience, respectively). Evaluations focused on signal intensity changes compared to pre-treatment, presence of inflammation and swelling post-treatment, and evidence of off-target muscle or skin damage. MRIs were additionally evaluated quantitatively using volumetric segmentations performed in the open-source software 3D Slicer (www.slicer.org). Segmentations were performed by the primary author as well as a medical student (A.S., 2 years of experience). Segmentations were performed by comparing pre- and post-treatment images slice-by-slice and highlighting on post-treatment imaging any areas with changes in signal intensity. Untreated tumor (“tumor”) and treated tumor (“treatment”) were segmented using a grow from seeds approach. Volume and mean signal intensity of the segmented tumor and ablation were calculated and averaged between observers.

Segmentations were performed separately on T2-weighted FS and T1-weighted FS CE images to allow for comparison between MRI sequences. For the latter, to normalize the signal intensity of the targeted tumor region of interest for each imaging session, a spherical volume (100–1000 voxels) was segmented in healthy muscle adjacent to the tumor. The spherical volume was chosen to be as close to the tumor and ablation (< 10 mm) as possible while selecting a homogenous region of muscle tissue. Normalized contrast enhancement was calculated by dividing the mean signal intensity of the unablated residual tumor by the mean signal intensity of the nearby healthy muscle.

### 2.4 Histopathology

Treated and untreated murine OS tumors were initially fixed in 10% formalin followed by decalcification in 10% EDTA until able to be easily, manually sectioned with a blade (approximately 3–5 days). Sections were then routinely embedded in paraffin, sectioned into 5 µm slices, and stained with hematoxylin and eosin (H&E) for histopathological analysis. Sections were assessed for the percentage of the tumor area exhibiting tumor necrosis, defined as the presence of dead or dying cells with varying amounts of eosinophilic debris and/or clumped basophilic nuclear debris with a variably intact stroma. A score of 0 indicates no evidence of cell death, 1 indicates up to 33% of the section exhibits features of cell death (mild), 2 indicates 33-66% of the section exhibits features of cell death (moderate), and 3 indicates more than 66% of the section exhibits features of cell death (marked). Sections were also assessed for the presence and relative amount of tumor hemorrhage which was defined as the presence of extravasated erythrocytes. This was scored 0–3 in a similar manner as tumor necrosis.

### 2.5 Histopathology versus MRI comparison

For a more thorough comparison of treatment outcomes between MRI and histopathology, for n = 4 mice, the formalin-fixed tumor-bearing limb was sliced and sectioned axially to match the post-treatment T2w FS MRI (n = 2 at 1 DPT, n = 2 at 7 DPT), which was performed immediately prior to sacrifice for comparison to histopathology. Histopathological characteristics of ablation (necrosis, hemorrhage) were scored as described above, and the same characteristics were scored on MRI by two human musculoskeletal radiologists. Intratumoral hemorrhage and necrosis were scored on T1- and T2-weighted MRI using a 4–point scale based on the estimated proportion of tumor volume involved (0 = none; 1 = mild, ≤ 33%; 2 = moderate, > 33–66%; 3 = severe, > 66%). Hemorrhage was defined as intralesional areas with intrinsic T1 hyperintense signal and corresponding T2 hypointensity or heterogenous, mixed signal consistent with blood products (with or without fluid-fluid levels). Necrosis was defined as fluid-like regions (marked T2-hyperintense/T1-hypointense) lacking internal solid components and showing no internal enhancement on post−contrast T1-weighted images. The two readers scored all cases independently, with disagreements resolved by discussion and subsequent consensus.

### 2.6 Statistical analysis

All statistical analyses were performed in GraphPad Prism (version 10.1.1) with statistical significance defined as *p* < 0.05. The residuals of all datasets being used for t-tests and ANOVAs were tested for normality using the Shapiro-Wilk, Anderson-Darling, Kolmogorov-Smirnov, and D’Agostino-Pearson tests; the residuals were considered to follow a normal distribution if *p* > 0.05 for at least 1 test. If the residuals did not follow a normal distribution (*p* < 0.05), the non-parametric statistical test equivalent was used. Summary statistics are reported and displayed as mean ± standard deviation. **** indicates *p* < 0.0001; *** indicates *p* < 0.001; ** indicates *p* < 0.01; * indicates *p* < 0.05.

T-tests were used to compare 1) targeted percent ablation between treated-small and treated-large tumor groups, and 2) tumor volume at time of histotripsy treatment between survival and untreated groups. Comparison of survival curves was performed using a Mantel-Cox log-rank test.

Pearson’s correlations were used to determine the relationships between 1) tumor volumes as measured on gross anatomy, US, and MR imaging; 2) tumor and ablation segmentation volumes on MRI between two observers; and 3) various tumor and ablation characteristics (pre-treatment tumor volume, percent ablation, remaining tumor volume, and ablated tumor volume) and survival. Variability between observers was considered within reason if there was a significant, positive correlation between observers (*p* < 0.05).

Repeated measures mixed-effects models with Tukey’s multiple comparisons were used to compare 1) MRI-segmented tumor volumes between treated-small, treated-large, and untreated mice at 1 and 7 DPT (as only treated-small tumors were able to survive and be imaged at 14 DPT, a t-test was used to evaluate treated-small tumor growth from 7 to 14 DPT); 2) T1w versus T2w tumor volumes over time, and 3) T1w versus T2w ablation volumes over time.

Volumes from each imaging sequence (T1w and T2w) were also evaluated using one-way ANOVAs with Tukey’s multiple comparisons (i.e. comparing treatment volume on T1w images over time). A one-way ANOVA with Tukey’s multiple comparisons was also used to evaluate tumor contrast enhancement on T1w MRI over time. A final one-way ANOVA was used to compare change in body weight, relative to pre-treatment body weight, for the first 5 days post-histotripsy. Lastly, two-way ANOVAs with Tukey’s multiple comparisons were used to compare histopathological versus MRI assessments of hemorrhage and necrosis over time, as well as comparing hemorrhage and necrosis scores on histopathology between the two treatment parameter sets.

## 3 Results

### 3.1 Tumor measurement modalities and calculation of percent ablation

In order to evaluate percent tumor ablation, a pre-treatment tumor volume needs to be calculated. For this study, tumor volumes were measured using 3 different modalities: manual caliper measurements based on gross anatomy, US imaging, and MR imaging. The average tumor volumes were as follows: 230 ± 104 mm^3^ from caliper measurements on the day of treatment; 138 ± 55 mm^3^ from US imaging on the day of treatment; and 216 ± 98 mm^3^ from MR imaging 24–48 hours before treatment. Additionally, the average tumor volume between multiple measuring modalities was calculated; calipers + US + MRI = 210 ± 77 mm^3^; and calipers + US = 206 ± 74 mm^3^). MRI has the best resolution of these modalities, and therefore tumor volumes from caliper only and US only measurements, as well as the average of caliper and US imaging measurements, were correlated against tumor volume on MRI to identify a secondary suitable tumor measurement modality (**Supplementary Figure 3**). The tumor volume averaged across US and gross caliper measurements had the strongest correlation to MRI-measured tumor volumes (*r* = 0.7933, R^2^ = 0.6293, *p* = <0.0001), and as such, percent ablation calculations use the averaged gross caliper and US tumor volume as the baseline pre-treatment tumor volume. MRI volumes are included in the pre-treatment tumor volume average when possible (i.e. not all mice were imaged with MRI due to study constraints).

### 3.2 Histotripsy outcomes and ultrasound imaging findings

Histotripsy ablation details are reported in **Table 1**. Mice were grouped according to tumor volume (calculated as described above) on the day of treatment, to create *treated-small* and *treated-large* groups. The average pre-treatment tumor volume, averaged across gross, US, and MRI measurements, was 143 ± 35 mm^3^ for treated-small tumors and 268 ± 44 mm^3^ for treated-large tumors. The average targeted ablation volume was 121 ± 60 mm^3^ for treated-small tumors and 173 ± 64 mm^3^ for treated-large tumors. When averaging the individual percent ablations across all mice per group, the targeted percent tumor ablation was similar between the two groups; 71 ± 19% for small tumors and 66 ± 24% mm for large tumors. The difference in targeted percent ablation between treated-small and treated-large groups was non-significant (*p* = 0.5570). Percent ablation is shown for all mice in **Supplementary Figure 4**. The average peak negative pressure at which treatment was applied was 33 ± 2–3 MPa for both tumor size groups (**Table 1**).

**Table 1:** Histotripsy treatment characteristics (mean ± standard deviation) across all 53 mice treated in the present study.

Treatment group	Treatment pressure (-MPa)	Treatment volume (mm^3^)	Tumor volume (mm^3^)	Percent ablation (%)	Percent swelling (%)
Small tumors (n = 34)	33 ± 3	121 ± 60	143 ± 35	71 ± 19	13 ± 8
Large tumors (n =19)	33 ± 2	173 ± 64	268 ± 44	66 ± 24	11 ± 7

**Figure 2:**
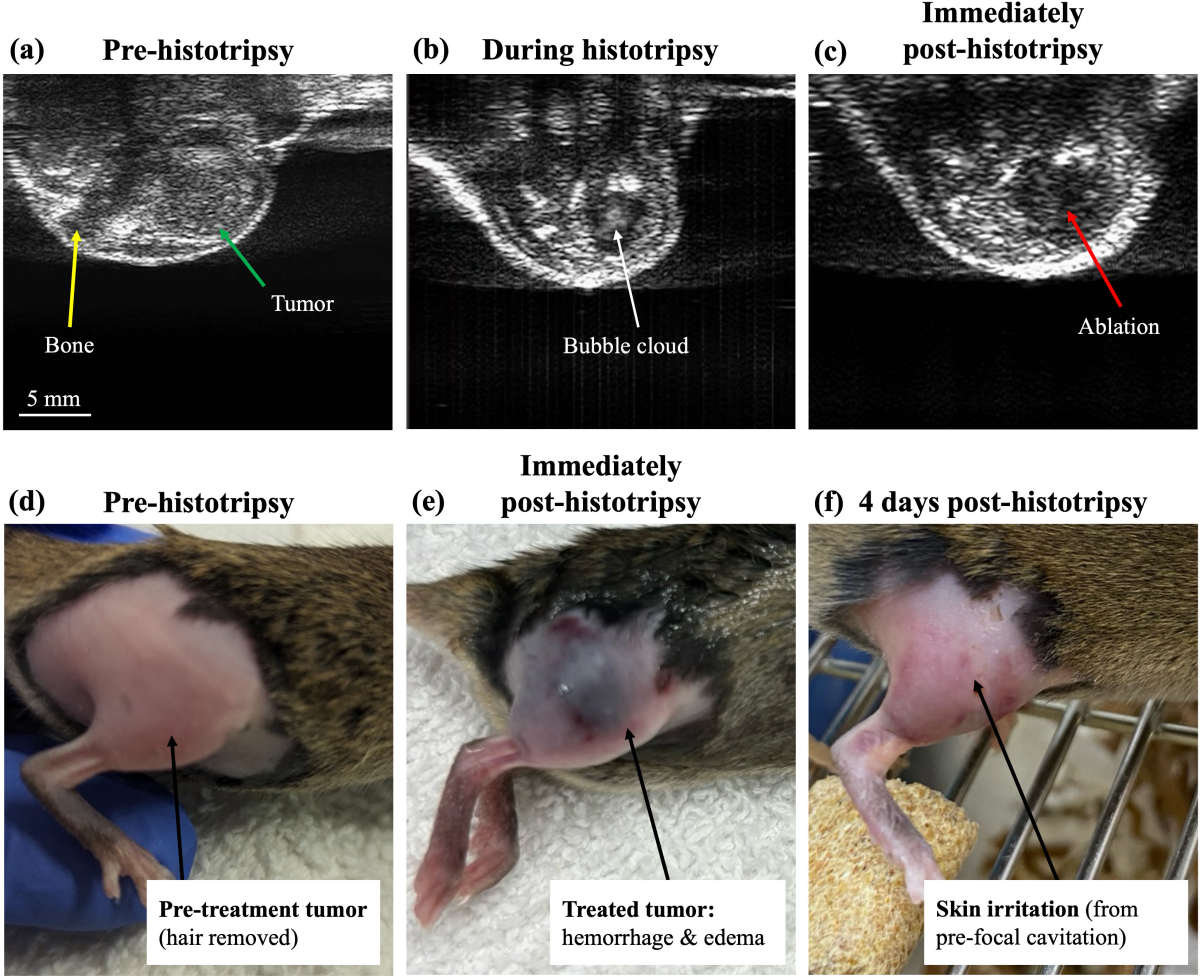
Before, during, and after histotripsy on real-time US imaging and on gross anatomy. **(a–c)** A representative orthotopic OS tumor in the right hindlimb on US imaging before **(a)**, during **(b)**, and after **(c)** histotripsy ablation. **(d–f)** Gross anatomy of a representative mouse with an orthotopic OS tumor, showing **(d)** tumor pre-treatment (8–12 days post-injection), **(e)** treated tumor immediately after histotripsy, and **(f)** treated tumor-bearing limb 4 days after histotripsy ablation.

Overall, mice tolerated histotripsy treatment well, and treatment duration was approximately 30–60 minutes depending on the volume targeted. Images of orthotopic OS tumors before and after histotripsy ablation are shown on US imaging and gross anatomy in **Figure 2**. Tumors were generally hyperechoic on real-time US imaging relative to the hypoechoic tibia (**Figure 2a**), and the hyperechoic oscillating bubble cloud was visible during treatment (**Figure 2b**). A video of this cavitation bubble cloud is shown in **Supplementary Figure 5**. There was a hypoechoic ablated area visible in the core of the tumor after histotripsy treatment (**Figure 2c**). **Figure 2d** shows a representative murine OS tumor on gross anatomy prior to histotripsy treatment. Immediately after treatment, the treated tumor was characterized by substantial bruising and mild to moderate skin trauma, along with swelling (**Figure 2e**). The swelling of the tumor and hypoechoic treatment zone was observable grossly on US, and as measured on US imaging, small tumors were 13 ± 8% larger immediately after treatment as compared to immediately before treatment, while large tumors were 11 ± 7% larger (**Table 1**). This swelling resolved grossly within 7 DPT. Mild to moderate skin irritation was observed in the majority of mice due to pre-existing skin trauma from the depilatory cream application and/or pre-focal cavitation on the skin, and this skin damage healed well over time (resolved within 4–7 DPT, **Figure 2f**). This swelling and skin irritation, along with other potential treatment effects, did not adversely affect body weight. Percent change in body weight for the first 5 DPT, relative to body weight on the day of treatment, is shown in **Supplementary Figure 6**; there was no significant effect of post-treatment timepoint on change in body weight [*p* = 0.4949, F(4,115) = 0.8524].

### 3.3 Histopathological assessment of histotripsy ablation

**Figure 3:**
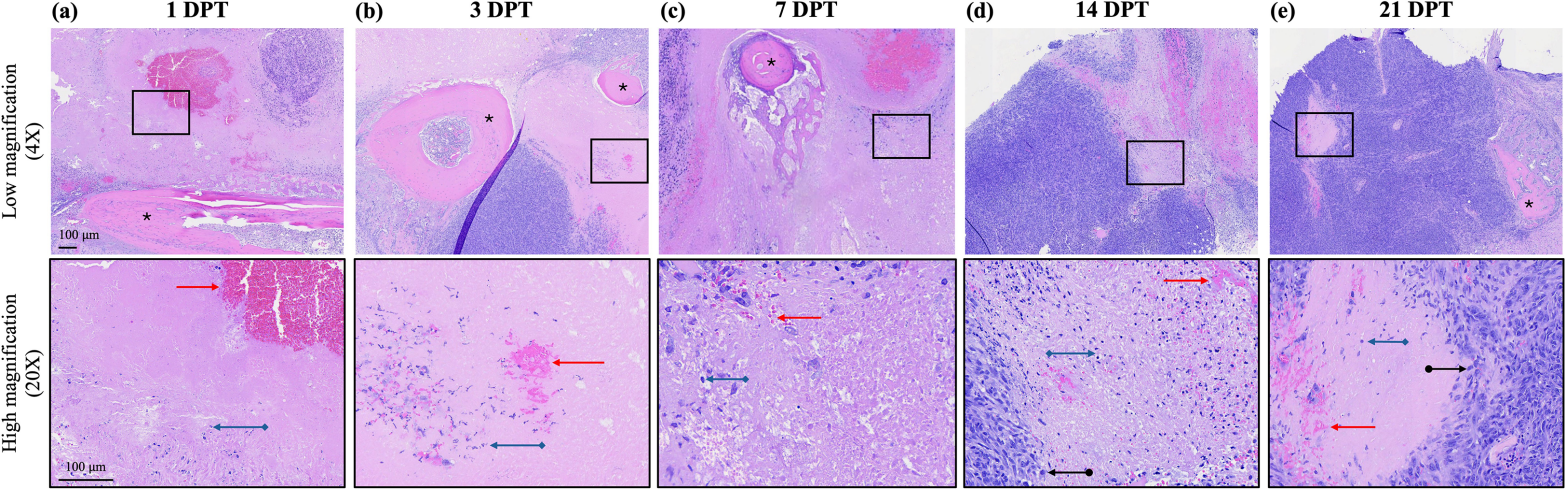
Representative low magnification (4X, top row) and high magnification (20X, bottom row) histopathology images at various days post-treatment (DPT). All images were routinely stained with H&E. **(a)** 1 DPT. **(b)** 3 DPT. **(c)** 7 DPT. **(d)** 14 DPT. **(e)** 21 DPT.

**Figure 4:**
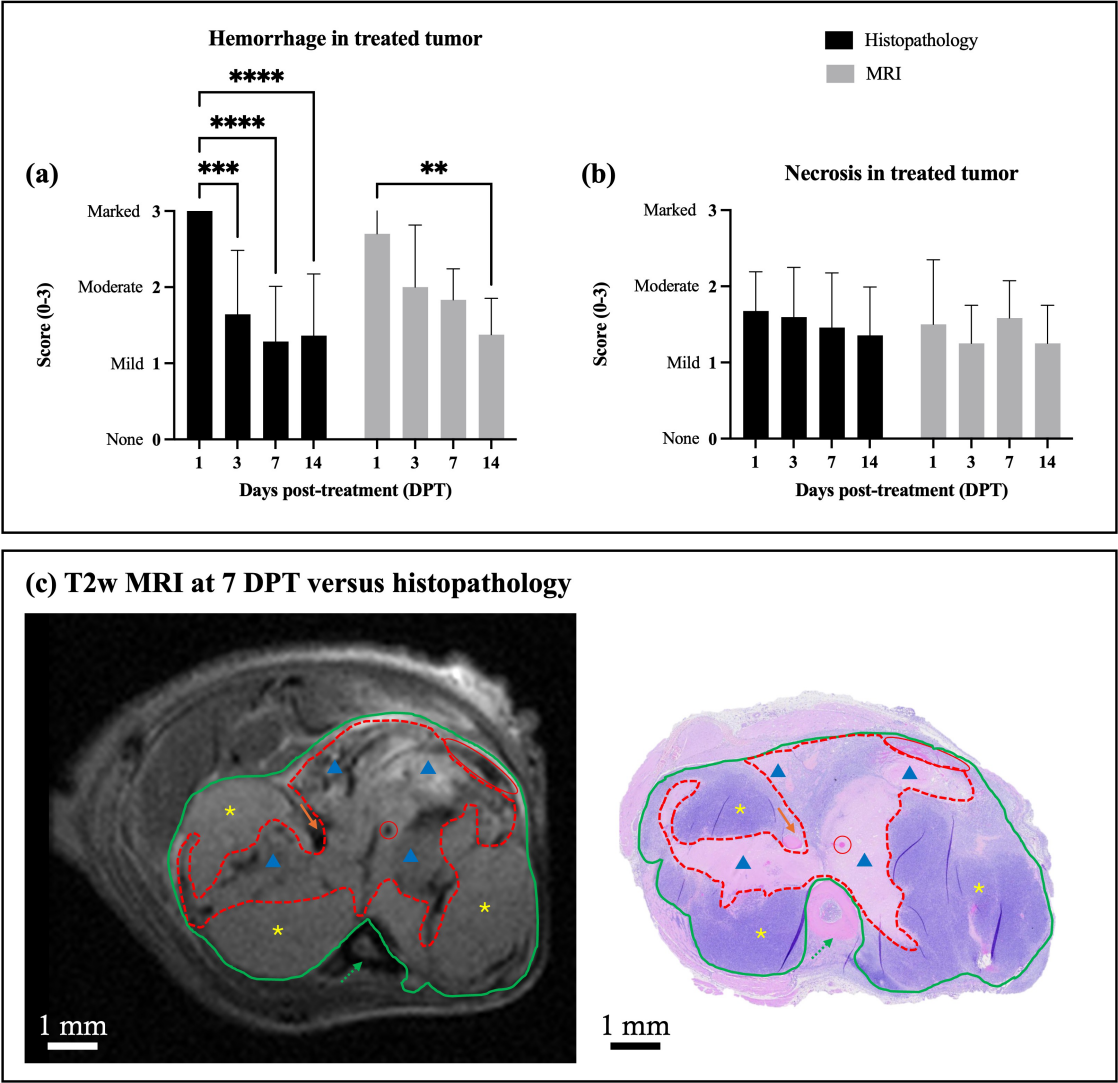
Semi-quantitative **(a, b)** and qualitative **(c)** comparisons of histotripsy ablation on MRI versus histopathology. For histopathological evaluations: 1 DPT, n = 8; 3 DPT, n = 14; 7 DPT, n = 14; 14 DPT, n = 11. For MR imaging evaluations: 1 DPT, n = 10; 3 DPT, n = 4; 7 DPT, n = 6; 14 DPT, n = 4. Significance was determined via two-way ANOVAs with Tukey’s multiple comparisons. ** indicates *p* < 0.005, *** indicates *p* < 0.0005, and **** indicates *p* < 0.0001. **(a)** Tumor hemorrhage over time, as quantified on MRI and histopathology. **(b)** Tumor necrosis over time, as quantified on MRI and histopathology. **(c)** Representative T2w MRI versus low-magnification histopathology images for a mouse with orthotopic OS at 7 DPT. Red circles indicate hemorrhage (within the remnants of a congested vessel); yellow asterisks indicate untreated healthy tumor; and blue triangles indicate the treatment zone. Dashed green arrows point to the tibia, while solid orange lines point to the fibula. The green solid outline details the tumor volume, while the red dashed outline details the treatment volume.

At the time of sacrifice, a portion of tumor was collected for gross and microscopic evaluation of ablation. Histopathological images are shown for representative animals sacrificed at 1, 3, 7, 14, and 21 DPT in **Figure 3**. Semi- quantitative scoring of histopathological features is shown in **Figure 4**. Qualitatively, the treatment is acutely characterized by a large acellular zone with regions of hemorrhage and occasional dead or dying (necrotic) OS cells. Both hemorrhage and necrosis were greater at acute timepoints than at later timepoints. Hemorrhage was visible at all post-treatment timepoints but was significantly greater at 1 DPT than at 3, 7, or 14 DPT (*p* < 0.05 for all comparisons, **Figure 4a**). There was a significant effect of timepoint on hemorrhage [*p* < 0.0001, F(3,62) = 14.68], with decreased hemorrhage at later timepoints. While there was no significant effect of timepoint on necrosis as seen on histopathology [*p* = 0.6963, F(3,62) = 0.4816], there was a trend that tumor necrosis decreased over time (**Figure 4b**). At later timepoints, starting at 7 DPT (**Figure 3c**), the treated area appears to begin the process of involution, and by 14 DPT (**Figure 3d**), the presumably residual untreated tumor becomes more apparent at the periphery of the treatment zone. Finally, by 21 DPT (**Figure 3e**), the treatment zone is nearly fully resorbed, with only a small acellular area corresponding to the treated tumor volume present.

### 3.4 Comparison of histotripsy outcomes on histopathology versus MRI

To validate the ablative effects as seen on MRI (reported below), histopathology of treated tumors was compared to imaging at 1, 3, 7, and 14 DPT, focusing on assessments of tumor hemorrhage and necrosis (**Figure 4**). Quantitatively, there were no significant difference in hemorrhage (**Figure 4a**) or necrosis (**Figure 4b**) scores measured on MRI or histopathology at any timepoint (*p* > 0.05 for all comparisons), indicating good agreement between modalities. Considering hemorrhage on MRI, there was a significant decrease in hemorrhage from 1 to 14 DPT (*p* = 0.0081), which matches well with the significant decrease in hemorrhage seen on histopathology from 1 DPT to 3, 7, and 14 DPT (**Figure 4a**). Considering necrosis, there were no significant effects or differences between MRI and histopathology and over time (*p* > 0.05 for all comparisons, **Figure 4b**), but there was a trend towards decreased necrosis over time, going from moderate to mild necrosis (**Figure 4b**).

Qualitatively, visualization of treated versus untreated tumor was clear between T2w MRI and histopathology (**Figure 4c**). Treated tumor areas, visible on histopathology as acellular debris with no intact tumor cells, were heterogeneously hypointense (more hemorrhagic) or hyperintense (more fluidic) on T2w MRI as compared to pre-treatment images. In contrast, untreated tumor had homogenous signal intensity on MRI (slightly hyperintense to muscle) and showed densely-packed areas of high cellularity on histopathology. The interface between treated and untreated tumor is precisely demarcated on histopathology, with necrotic tumor cells and tumor cell debris evident, while the treatment interface is less obvious on MRI, showing subtle to obvious differences in signal intensity between treated (heterogeneous signal) and untreated (homogenous signal) tumor (**Figure 4c**).

### 3.5 Tumor and treatment signal characteristics over time on MRI

In order to ensure accurate identification and segmentation of murine OS tumors and histotripsy-treated zones, tumor and treatment volumes were segmented by two observers on T2w and T1w FS MRI. Individual observer measurements show a significant positive correlation (*p* < 0.05) for tumor segmentations from untreated and pre-treatment images, as well as for both tumor and treatment segmentations from acute (1 DPT) and sub-acute (7 and 14 DPT) images. Correlation plots are shown in **Supplementary Figure 7**, indicating good agreement between observers and allowing for further imaging analysis.

**Figure 5:**
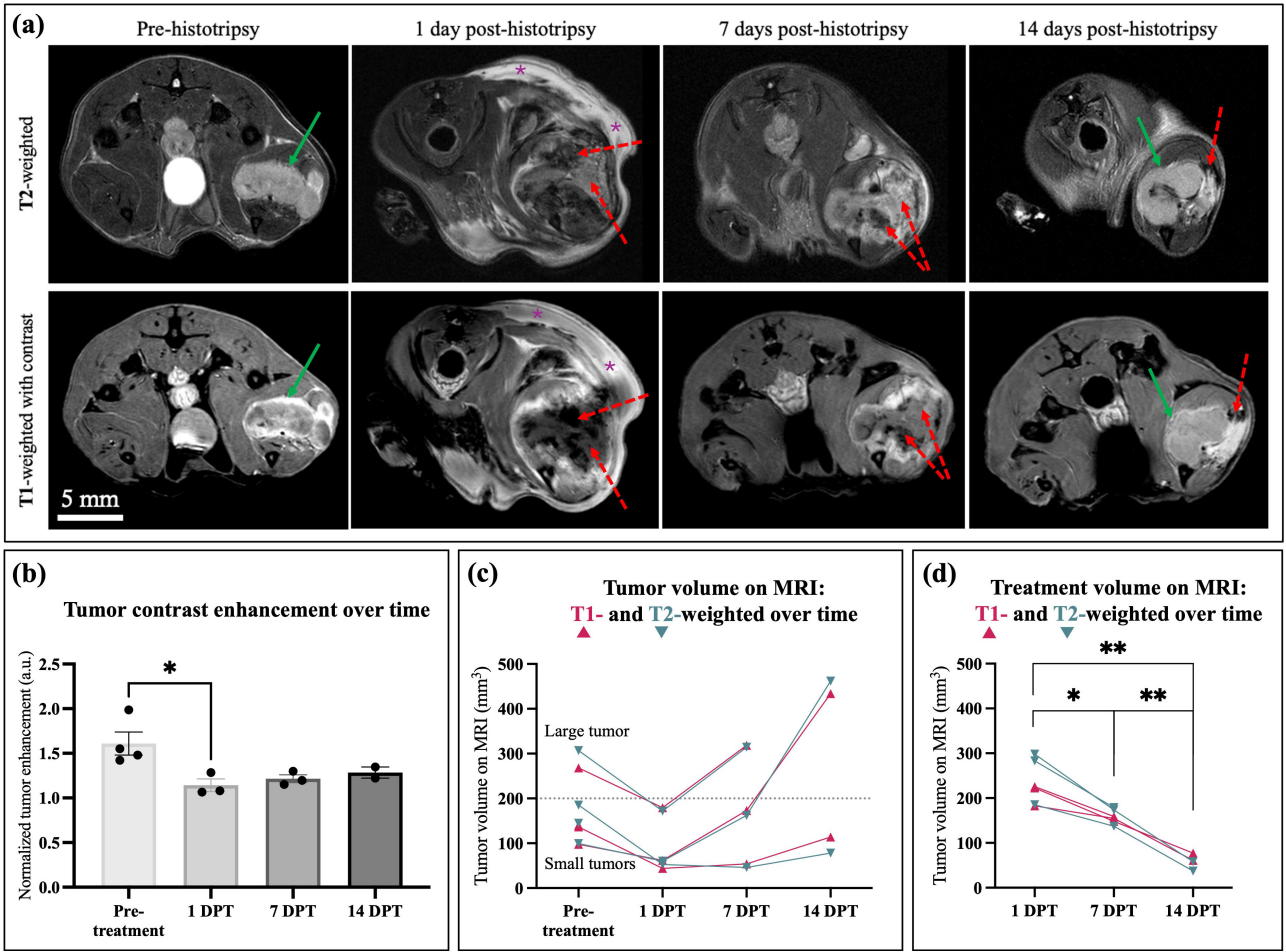
Axial T2-weighted vs. T1-weighted contrast-enhanced MRI over time before and after histotripsy treatment in murine orthotopic OS. **(a)** Representative images of a mouse with orthotopic OS before and after histotripsy treatment, as shown on T1w and T2w MRI. **(b)** Whole tumor contrast enhancement on T1w MRI before and over time after histotripsy ablation. Significance was determined via a one-way ANOVA with Tukey’s multiple comparisons. **(c)** Tumor volume on T1w versus T2w images from orthotopic OS mice treated with histotripsy and imaged with MR. The dashed horizontal line indicates the threshold of pre-treatment tumor volume for classification as treated-large (> 200 mm^3^) or treated-small (< 200 mm^3^). **(d)** Treatment volume on T1w versus T2w images, showing significant treatment zone resorption over time. Significance was determined via repeated measures mixed-effects models with Tukey’s multiple comparisons. DPT = days post-treatment. * indicates *p* < 0.05, and ** indicates *p* < 0.005.

Representative T1w versus T2w images for a single mouse are shown in **Figure 5a**. On T2w FS MR images, the pre-treatment tumor was hyperintense relative to healthy muscle. The tumor was often interfacing with the tibial bone surface but occasionally had muscle between the tumor and bone at some points along the length of the bone. 1 DPT, the treatment zone is characterized primarily by heterogeneous T2-hypointensity (likely hemorrhage) at the ablation core, with occasional hyperintensity (likely edema and/or residual tumor) at the rim or periphery of the ablated tumor. There was commonly a layer of hyperintensity underneath the skin, indicating edema and inflammation from the treatment. At 7 DPT, the ablation volume had resorbed and appeared smaller but was still readily identifiable. At this timepoint, the treated tumor was primarily T2-hyperintense, which corresponded to non-enhancement on T1-weighted imaging, with some hypointensity (blood products) remaining at the ablation core. By the latest 14 DPT timepoint, the treatment zone was nearly full resorbed, and any presumably residual untreated tumor (enhancing) had begun to proliferate at the periphery of the treatment area.

T1w images matched well with T2w assessments of ablation. The area of hemorrhage (T2-hypointensity) and fluid (T2-hyperintensity) seen as T2w MRI was non-enhancing (presumably non-viable) on T1w CE MRI. Qualitatively, on T1w images, the ablation volume was characterized by a lack of contrast enhancement in the ablation core, with a rim of hyperintense inflammation and/or residual tumor surrounding the targeted tumor. There was a significant decrease in contrast enhancement of the whole tumor from pre-treatment to 1 DPT (34% decrease, *p* = 0.0480, **Figure 5b**), suggesting decreased tumor viability and increased necrosis immediately after histotripsy treatment. Tumor contrast enhancement remained lower than pre-treatment levels at 7 and 14 DPT (28 and 22% lower than baseline, respectively).

Quantitatively, tumor volume for the 3 mice (1 treated-large, 2 treated-small) imaged with both MRI sequences was compared on T1w versus T2w images in **Figure 5c**. There were no significant differences in tumor volume as measured on T1w versus T2w MR images (*p* > 0.05 for all comparisons). The treated-large tumor showed a 2% increase in size from pre-treatment to 7 DPT, while the 2 treated-small tumors showed an average of 27% decrease in size at the same time point. The treated-large mouse was unable to survive to 14 DPT, while both treated-small tumors were imaged at this timepoint. Within the treated-small tumors, 1 mouse showed tumor enlargement over time (366% increase from pre-treatment to 14 DPT), while the other showed tumor stabilization (58% decrease from pre-treatment to 14 DPT).

### 3.6 Tumor and treatment volumes over time on MRI

Ablation volume was segmented and compared between T1w versus T2w images (**Figure 5d**). There were no significant differences between ablation volume on T1w versus T2w MR images (*p* > 0.05 for all comparisons). Overall, the ablation volume (averaged between T1w and T2w images) showed significant resorption over time. Ablation volumes decreased significantly from 1 to 7 DPT (32% decrease, *p* = 0.0192) and even further from 7 to 14 DPT (63% decrease, *p* = 0.0055). In total, ablation volume decreased significantly from 1 to 14 DPT (75% decrease, *p* = 0.0005). By 14 DPT, the treatment zone had largely disappeared but was identifiable by lingering hyperintensity in the area.

**Figure 6:**
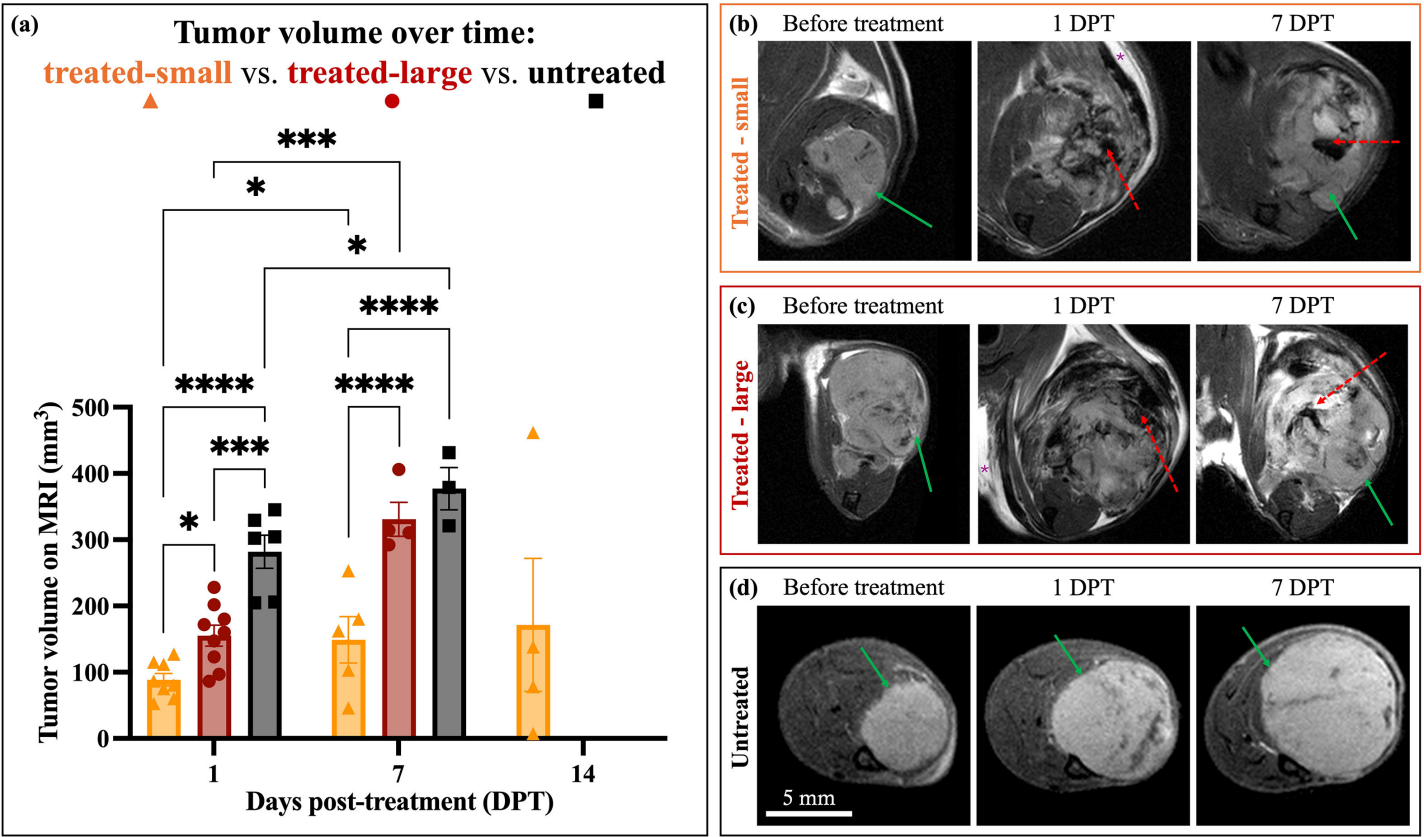
Axial T2-weighted MRI before and over time after histotripsy treatment in treated versus untreated orthotopic OS mice. Tumors are indicated with green solid arrows, while treatment zones are identified with red dashed arrows. Edema is indicated with a yellow asterisk. DPT = days post-treatment. **(a)** Segmented tumor volumes on MRI in treated-small, treated-large, and untreated OS mice before treatment and at 1 and 7 DPT. Treated-small is indicated by orange triangles, treated-large is indicated by red circles, and untreated is indicated by black squares. Significance between 1 and 7 DPT was determined via repeated measures mixed-effects models with Tukey’s multiple comparisons. Significance between treated-small tumor volume at 7 and 14 DPT was determined via Mann-Whitney test (p = 0.7302, non-significant). * indicates *p* < 0.05, ** indicates *p* < 0.005, *** indicates *p* < 0.0005, and **** indicates *p* < 0.0001.**(b)** Representative MR images from a treated-small murine OS tumor before treatment and at 1 and 7 DPT. **(c)** Representative MR images from a treated-large murine OS tumor before treatment and at 1 and 7 DPT. **(d)** Representative MR images from an untreated murine OS tumor over time, for comparison to the treated DPT.

Tumor volume quantification over time focused on T2w images, as these were acquired for all mice imaged. Tumor volumes on MRI were compared between treated-small and treated-large tumors, as well as compared to untreated tumors at similar days post-tumor cell injection (**Figure 6a**). Quantitatively, there was a significant effect of treatment group [treated-small, treated-large, and untreated; *p* < 0.0001, F(2,21) = 37.74] and timepoint [1 DPT and 7 DPT; *p* = 0.0001, F(1,8) = 47.67], as well as a significant interaction between treatment group and timepoint [*p* = 0.0494, F(2,8) = 4.486]. Treated-small tumors were significantly smaller than untreated tumors at 1 (74% decrease, *p* < 0.0001) and 7 (64% decrease, *p* < 0.0001) DPT, while treated-large tumors were only significantly smaller than untreated tumors at 1 DPT (47% decrease, *p* = 0.0002). Additionally, treated-small tumors were significantly smaller than treated-large tumors at 1 (51% decrease, *p* = 0.0362) and 7 (57% decrease, *p* < 0.0001) DPT. The only comparison that was not significant was between treated-large tumors and untreated tumors at 7 DPT (*p* = 0.1934). All groups had significant tumor progression between 1 and 7 DPT (*p* < 0.05 for all comparisons).

Mice with treated-small tumors were able to survive up to and beyond 14 DPT, allowing for data at further post-treatment timepoints (**Figure 6a**). There was no significant increase in treated-small tumor volume from 7 to 14 DPT (23% increase, *p* = 0.7302). Generally, while the treated-large and untreated tumors continued to grow between each timepoint, treated-small tumors were relatively stable over time. Examples of treated versus untreated tumors on T2w FS MRI are shown in **Figure 6b** (treated-small), **Figure 6c** (treated-large), **Figure 6d** (untreated).

### 3.7 Survival after histotripsy treatment

**Figure 7:**
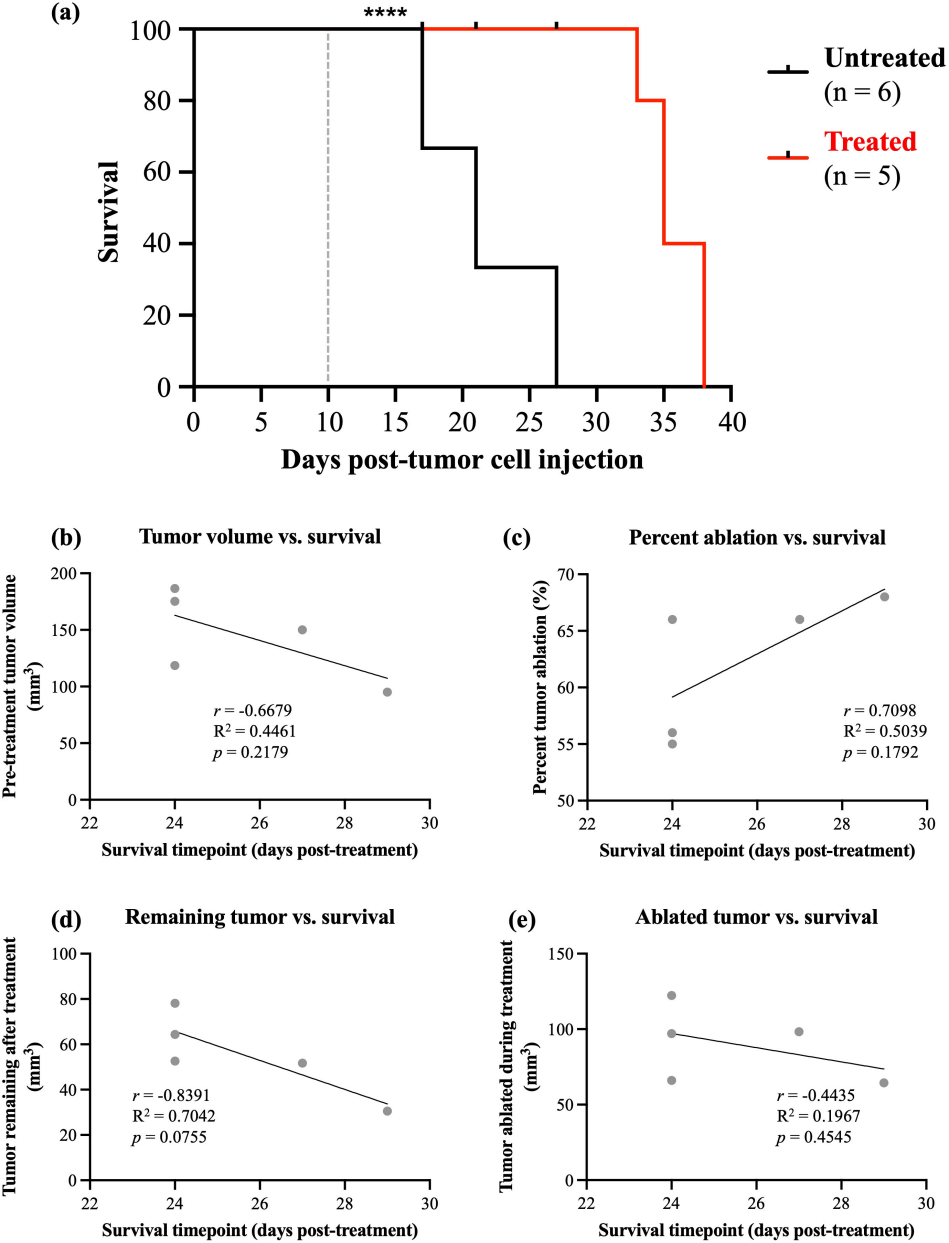
Survival data and correlating factors from orthotopic OS mice treated with histotripsy. **(a)** Kaplan-Meier survival curve for histotripsy-treated versus untreated OS mice. Dashed line indicates approximate timepoint of histotripsy treatment. **** indicates *p* < 0.0001. **(b)** Pearson’s correlation between tumor volume at time of treatment versus survival. **(c)** Pearson’s correlation between percent ablation (volume of tumor ablated/pre-treatment tumor volume) versus survival. **(d)** Pearson’s correlation between remaining tumor volume (pre-treatment tumor volume – volume of tumor ablated) versus survival. **(e)** Pearson’s correlation between ablated tumor volume versus survival.

Survival beyond 14 DPT was evaluated in a group of 5 mice survived, and sacrificed when they met endpoint criteria or at the predetermined study endpoint of 30 DPT. Only treated-small mice, not treated-large or untreated, were able to survive beyond 14 DPT. At the time of histotripsy treatment, the average tumor volume for the mice in the survival group (all treated-small) was 190 ± 47 mm^3^, and the average tumor volume for untreated mice at this time was 211 ± 23 mm^3^. There was no significant difference in tumor volumes at time of histotripsy treatment between the survival and untreated groups (*p* = 0.2978, data not shown). Survival data between treated-small and untreated mice are shown in **Figure 7a**. Histotripsy-treated mice lived significantly longer than untreated mice (*p* < 0.0001). Treated mice had a median survival of 35 days post-tumor cell injection (~25 DPT), while untreated mice had a median survival of 20 days post-tumor cell injection. The longest survival of a treated mouse was 39 days post-tumor cell injection (equating to 30 DPT), and this mouse was euthanized due to the predetermined study endpoint time (30 DPT).

Correlations were performed to evaluate how ablative factors impact survival (**Figure 7**). Factors evaluated were pre-treatment tumor volume, percent ablation, remaining tumor (non-targeted and untreated residual tumor), and ablated tumor (volume of tumor treated). While none of these factors were statistically significantly correlated with survival, the trends shown warrant further investigation. Pre-treatment tumor volume did not significantly correlate with survival (*r* = -0.6679, R^2^ = 0.4461, *p* = 0.2179, **Figure 7b**), although only treated-small mice were observed to survive beyond 14 DPT. Percent ablation had a strong but not significant positive correlation with survival (*r* = 0.7098, R^2^ = 0.5039, *p* = 0.1792, **Figure 7c**). Similarly, remaining tumor (i.e. non-ablated tumor) had a strong negative correlation with survival that was nearing statistical significance (*r* = 0.8391, R^2^ = 0.7042, *p* = 0.0755, **Figure 7d**). There was no significant correlation between ablated tumor volume and survival (*r* = -0.4435, R^2^ = 0.1967, *p* = 0.4545, **Figure 7e**).

One mouse in the survival group (30 DPT survival) had no evidence of neoplastic tumor tissue on histopathology after sacrifice. This mouse had the highest percent ablation (68%) of the survival group, which had an average percent ablation of 62 ± 6%. On the day of treatment, this mouse also had the smallest tumor (95 mm^3^) of the survival group, which had an average tumor size of 145 ± 38 mm^3^ on treatment day (similar to the average tumor size of the entire treated-small group). A total of 4 mice in the entire study of 53 mice had tumor volumes under 100 mm^3^ (7.5% of all mice), this includes the one mouse in the survival group that showed complete tumor regression.

## 4 Discussion

To our knowledge, this is the first study to evaluate the long-term ablative outcomes after histotripsy ablation in a syngeneic orthotopic mouse model of OS. This study is important to expand our knowledge on the long-term outcomes of histotripsy ablation in OS, as well as to increase the utility and biological relevance of this preclinical murine model for histotripsy evaluations. We found that histotripsy treatment significantly reduced tumor burden and improved survival as compared to untreated mice. Histotripsy treatment resulted in edema-induced swelling and inflammation, which was evident grossly and on US and MR imaging. Resolution of treatment zones began by 7 DPT based on MRI and was nearly complete by 14–21 DPT. This corroborates with prior research showing resolution of liver ablation within 1 month in murine models with similar ablation volumes to those in this study (24, 39). Prior work in large animals (30, 54) and human clinical trials (48, 49) has shown similar lesion resorption after histotripsy in the months following histotripsy, with the time to near complete resolution being dependent on the size of the ablated volume.

Another major goal of this study was to assess the evolution of the histotripsy-treated tumor on MR imaging, validated by histopathology. MRI has previously been used for a preliminary acute assessment of histotripsy ablation in orthotopic OS mice treated with histotripsy, showing T2w-hypointensity (hemorrhage), but no images have characterized ablation beyond 1 DPT (51). Beyond ablative assessments, MRI has rarely been utilized in murine orthotopic OS, with its use limited to validating tumor presence in novel model development (59, 60). MRI has been more widely used for preclinical histotripsy ablation assessment in soft tissues, such as orthotopic brain (44) and liver (39, 40) tumor mouse models. Previously reported findings from liver and brain tumors support the findings in the current study, showing an acutely T2-hypointense histotripsy treatment zone (likely reflecting necrosis and blood products) with increasing hyperintensity (likely edema and fluid) by 7 DPT and beyond (39, 40).

Studies in the liver have also focused on T2w MRI, as was the focus of this study’s MR imaging, but studies in the brain have explored CE MRI. Histotripsy-treated brain tumor models show similar CE imaging trends as seen in our OS mice, with decreased enhancement immediately post-ablation and increasing residual enhancement over time after treatment (44). Residual tumor growth was evident by 14 DPT in orthotopic OS as well as in previously reported brain (44) and liver tumors (39, 40). Overall, the MRI data presented in the current study matches well with previously reported preclinical rodent investigations of histotripsy ablation in other tissues, suggesting that different soft tissue tumors may respond similarly to histotripsy ablation on MRI, at least in the context of preclinical rodent models. This finding is attributed to the predominant soft tissue component of the murine OS model, and since canine and human OS can be more heterogeneous with more bone involvement, additional work is warranted to optimize MRI methods across the range of OS that presents clinically.

In humans with OS, MRI is the most common imaging modality for assessing tumor stage and progression, as MRI can capture the soft tissue extension of the tumor (61–64). In veterinary medicine, this imaging modality is used less commonly for OS, but a recent study led by our group demonstrated the utility of MRI for the acute assessment of histotripsy ablation in canine OS (21). In particular, T1w images with contrast were most useful in identifying and assessing the histotripsy treatment zone, whereas T2w images were only helpful for ablation targeted in extracortical or softer regions of tumor. The current study found that in murine OS, ablation was clear on both T2w and T1w CE MRI, likely attributed to the more homogeneous and softer nature of the murine tumor model allowing for the fluid-sensitive T2 sequence to better visualize ablative effects. On CE T1w after histotripsy ablation, murine OS behaved similarly to canine OS and showed an acute significant decrease in contrast enhancement. The immediate decrease in contrast enhancement suggests an acute decrease in tumor vascularization and viability, with the potential for sustained tumor necrosis over time. This effect is similar to the decrease in enhancement seen in human OS after treatment with radiation and chemotherapy, and the lack of enhancement has been validated on histopathologically to correlate to tumor necrosis (65).

Histopathologic validation of imaging findings is especially important when using an imaging modality to assess a novel treatment response. In this study, semi-quantitative assessments of hemorrhage and necrosis over time using both histopathology and MRI were well-matched and showed similar trends. Additionally, localization of treatment damage on both histopathology and MRI was also well-matched, and visualization of key features such as hemorrhage was present and in agreement between MRI and histopathology. Microscopically, ablation was characterized by an acellular treatment zone with acute hemorrhage, with scattered dead or dying OS cells and debris. This hemorrhage was well-visualized on MRI as T2-hypointensity. The interface between treated and untreated tumor was more obvious on histopathology than on MRI, but the overall shape and location of the treatment zone was apparent across modalities. Overall, this work validates the use of MRI to assess and monitor histotripsy treatment in an orthotopic OS murine model, which builds the potential for future studies to follow longer-term outcomes without the need for histopathological verification of ablation.

Another important finding from this work is that treating mice earlier in disease progression, when the tumor was smaller, had better outcomes than treating mice later in disease progression, when the tumor was larger. While mice were treated in the same timeframe (8–12 days) post-injection of tumor cells, some tumors grew more quickly than others and were therefore larger at the time of treatment. In the World Health Organization’s classification of tumor staging, size is a primary factor that contributes to tumor stage, and therefore we evaluated differences in tumor size at time of treatment. While tumor volume itself did not have a significant correlation with survival in our murine study, mice in the treated-large group (pre-treatment tumor volume > 200 mm^3^) met endpoint criteria before reaching the 14**+** DPT threshold, so only treated-small mice are in the survival group. This agrees with previous evidence in pediatric OS patients showing that early disease progression before treatment is associated with poor prognosis (66). Additionally, we found that the amount of tumor remaining (i.e. presumed non-ablated tumor) had a strong but not significant negative correlation with survival, while percent ablation had a strong but not significant positive correlation with survival. Together, these data suggest that the more tissue is ablated in a small tumor, the better the outcome. This is further validated by the fact that the one mouse that showed complete tumor regression in this study had the smallest tumor and the highest percent ablation of the survival group. Altogether, the results from this pilot study suggest that earlier and more complete ablation of murine OS tumors leads to better clinical outcomes, and further investigation with larger groups is warranted.

Future directions for this work include developing higher frequency histotripsy devices for more precisely targeting murine tumors. This would allow for more complete ablation, especially enabling treatment immediately below the skin without inducing prefocal cavitation. Skin damage was noted after most histotripsy treatments in this study and therefore limited the safely attainable percent ablation, but our data suggests that achieving a higher percent ablation yields the best results. Prior research using histotripsy to ablate orthotopic liver tumors has shown that achieving > 50% ablation is sufficient for inducing complete tumor regression in 80% of rats, while < 25% ablation resulted in eventual tumor progression (39, 40). It is worth noting that the tumor volumes reported in one of these studies (40) were 86.25 ± 15.12 mm^3^, whereas our treated-small tumors were 143 ± 35 mm^3^. It is also worth noting that the larger size of rats (~200 g) as compared to mice (~ 20 g) allows for greater precision of the 1 MHz bubble cloud and therefore the potential for more complete tumor ablation. Additionally, the liver is situated deep in the body, away from the superficial skin surface. There are also inherent differences in the tumor biology and microenvironment of bone and liver tumors, such that each tumor type might respond differently to histotripsy and require different parameters for the optimal ablation response. It is possible that for tumors such as OS, combination therapies are needed to reliably achieve complete tumor regression without metastatic disease in cases where the entire tumor volume cannot be targeted. The overall goal of this work is to develop histotripsy as a non-invasive limb-sparing treatment option for canine and human OS, and future work will translate these preclinical findings into the clinical setting.

## 5 Conclusions

This is the first study to evaluate the long-term effects of histotripsy ablation in a syngeneic orthotopic murine model of OS and to thoroughly compare MRI and histopathological results after histotripsy ablation. All findings on MR imaging were validated on histopathology and showed no significant differences between assessments, demonstrating the utility of MRI for monitoring tumor and ablation in murine orthotopic OS. This study also establishes the use of this orthotopic murine model for long-term histotripsy evaluations, allowing for future studies to further explore the preclinical potential of histotripsy for OS even beyond 30 DPT. Overall, histotripsy treatment significantly reduced tumor burden and tumor viability in the acute setting, with long-term significant benefits to survival. Tumors treated when small had better outcomes than tumors treated when large and only treated-small mice were able to survive beyond 14 DPT (treated-large and untreated mice reached endpoint criteria by that timepoint). This, in combination with our data showing a strong positive correlation between percent ablation and survival and a strong negative correlation between remaining tumor volume and survival, suggests that the best clinical outcomes occur when smaller tumors are ablated more completely, or when less tumor volume is remaining after ablation. Together, these results of this pilot study highlight the promise of histotripsy as a non-invasive treatment for OS, and this study shows the first long-term evidence of survival benefit and the potential for tumor regression in a preclinical model of OS.

## Data Availability

The raw data supporting the conclusions of this article will be made available by the authors, without undue reservation.

## Supplementary material

Supplementary Figure 1 Qualitative **(a–b)** and quantitative **(c–d)** comparisons between the two different treatment paradigms used in this study. For qualitative data, all histopathology images were routinely stained with H&E; red solid arrows indicate hemorrhage, while green dashed arrows indicate necrotic tumor cells. For quantitative data, two-way ANOVAs with Tukey’s multiple comparisons were used to assess statistical significance for hemorrhage and necrosis as scored on histopathology; * indicates *p* < 0.05, and ** indicates *p* < 0.005. Blue circles indicate the 1000 ppl at 1.5 x 0.75 x 0.75 mm spacing, while pink triangles indicate the 500 ppl at 1.0 x 0.5 x 0.5 mm spacing. **(a)** Representative histopathology at 10X magnification of the ablation zone at 7 DPT for the 1000 ppl at 1.5 x 0.75 x 0.75 mm spacing group. **(b)** Representative histopathology at 10X magnification of the ablation zone at 7 DPT for the 500 ppl at 1.0 x 0.5 x 0.5 mm spacing group. **(c)** Hemorrhage as scored on histopathology between the two treatment paradigms. **(d)** Necrosis as scored on histopathology between the two treatment paradigms.

Supplementary Figure 2 T1-weighted MR images of a representative mouse with OS before **(b)** and at different timepoints **(c, d)** post-injection of gadolinium-based contrast, with comparison to T2-weighted MRI **(a)**.

Supplementary Figure 3 Pearson’s correlation of actual tumor volume, as segmented on MRI, compared to tumor volumes measured from gross anatomy, US imaging, and an average of the two.

Supplementary Figure 4 Percent tumor ablation between treated-small and treated-large treatment groups. There was no significant difference in percent ablation between treatment groups (*p* = 0.5570, t-test).

Supplementary Figure 5 Real-time US imaging of a representative murine OS tumor being treated with histotripsy. The bubble cloud is visible as hyperechoic, oscillating structure in the core of the tumor.

Supplementary Figure 6 Percent change in body weight, relative to pre-treatment weight, for the first 5 days post-histotripsy. There was no significant effect of post-treatment timepoint on change in body weight (*p* = 0.4949, one-way ANOVA).

Supplementary Figure 7 Pearson’s correlations of MRI tumor and treatment zone segmentations from two individual observers. **(a)** Pre-treatment tumor segmentations. **(b)** 1 day post-treatment tumor segmentations. **(c)** 1 day post-treatment treatment segmentations. **(d)** Untreated tumor segmentations. **(e)** 7 and 14 days post-treatment tumor segmentations. **(f)** 7 and 14 days post-treatment treatment segmentations.

## References

[1] Durfee RA Mohammed M Luu HH . Review of osteosarcoma and current management. Rheumatol Ther. (2016) 3:221–43. doi: 10.1007/s40744-016-0046-y, PMID: 27761754 PMC5127970

[2] Misaghi A Goldin A Awad M Kulidjian AA . Osteosarcoma: a comprehensive review. SICOT-J. (2018) 4:12. doi: 10.1051/sicotj/2017028, PMID: 29629690 PMC5890448

[3] Mirabello L Troisi RJ Savage SA . International osteosarcoma incidence patterns in children and adolescents, middle ages and elderly persons. Int J Cancer. (2009) 125:229–34. doi: 10.1002/ijc.24320, PMID: 19330840 PMC3048853

[4] Mirabello L Troisi RJ Savage SA . Osteosarcoma incidence and survival rates from 1973 to 2004: Data from the Surveillance, Epidemiology, and End Results Program. Cancer. (2009) 115:1531–43. doi: 10.1002/cncr.24121, PMID: 19197972 PMC2813207

[5] Mueller F Fuchs B Kaser-Hotz B . Comparative biology of human and canine osteosarcoma. Anticancer Res. (2007) 27:155–64. 17352227

[6] Tarone L Mareschi K Tirtei E Giacobino D Camerino M Buracco P . Improving osteosarcoma treatment: comparative oncology in action. Life. (2022) 12:2099. doi: 10.3390/life12122099, PMID: 36556464 PMC9783386

[7] Savage SA Mirabello L . Using epidemiology and genomics to understand osteosarcoma etiology. Sarcoma. (2011) 2011:548151. doi: 10.1155/2011/548151, PMID: 21437228 PMC3061299

[8] Anfinsen KP Grotmol T Bruland OS Jonasdottir TJ . Breed-specific incidence rates of canine primary bone tumors — A population based survey of dogs in Norway. Can J Vet Res. (2011) 75:209–15. PMC3122972 22210997

[9] Simpson S Dunning MD de Brot S Grau-Roma L Mongan NP Rutland CS . Comparative review of human and canine osteosarcoma: morphology, epidemiology, prognosis, treatment and genetics. Acta Vet Scand. (2017) 59:71. doi: 10.1186/s13028-017-0341-9, PMID: 29065898 PMC5655853

[10] Qureshi MK Ghaffar A Tak S Khaled A . Limb salvage versus amputation: A review of the current evidence. Cureus. (2020) 12:e10092. doi: 10.7759/cureus.10092, PMID: 33005513 PMC7522192

[11] Jeys LM Kulkarni A Grimer RJ Carter SR Tillman RM Abudu A . Endoprosthetic reconstruction for the treatment of musculoskeletal tumors of the appendicular skeleton and pelvis. JBJS. (2008) 90:1265. doi: 10.2106/JBJS.F.01324, PMID: 18519320

[12] Smolle MA Leithner A Kapper M Demmer G Trost C Bergovec M . Complications, mobility, and quality of life in ankle sarcoma patients: any difference in limb salvage versus amputation? Bone Jt J. (2021) 103-B:553–61. doi: 10.1302/0301-620X.103B3.BJJ-2020-1308.R1, PMID: 33641415

[13] Grimer RJ . Size matters for sarcomas! Ann R Coll Surg Engl. (2006) 88:519–24. doi: 10.1308/003588406X130651, PMID: 17059708 PMC1963770

[14] Smith G Johnson G Grimer R Wilson S . Trends in presentation of bone and soft tissue sarcomas over 25 years: little evidence of earlier diagnosis. Ann R Coll Surg Engl. (2011) 93:542–7. doi: 10.1308/147870811X13137608455055, PMID: 22004638 PMC3604925

[15] Smeland S Bielack SS Whelan J Bernstein M Hogendoorn P Krailo MD . Survival and prognosis with osteosarcoma: outcomes in more than 2000 patients in the EURAMOS-1 (European and American Osteosarcoma Study) cohort. Eur J Cancer. (2019) 109:36–50. doi: 10.1016/j.ejca.2018.11.027, PMID: 30685685 PMC6506906

[16] Odri GA Tchicaya-Bouanga J Yoon DJY Modrowski D . Metastatic progression of osteosarcomas: A review of current knowledge of environmental versus oncogenic drivers. Cancers. (2022) 14:360. doi: 10.3390/cancers14020360, PMID: 35053522 PMC8774233

[17] Mehrvar A Mehrvar N Sadeghi Y Tashvighi M . Outcomes and survival rates of childhood osteosarcoma in Iran, A report from MAHAK Pediatric Cancer Treatment and Research Center, from 2007 to 2020. J Cancer Res Ther. (2023) 19:S272. doi: 10.4103/jcrt.JCRT_1559_20, PMID: 37148004

[18] Arnold L Hendricks-Wenger A Coutermarsh-Ott S Gannon J Hay AN Dervisis N . Histotripsy ablation of bone tumors: feasibility study in excised canine osteosarcoma tumors. Ultrasound Med Biol. (2021) 47:3435–46. doi: 10.1016/j.ultrasmedbio.2021.08.004, PMID: 34462159 PMC8578360

[19] Ruger LN Hay AN Gannon JM Sheppard HO Coutermarsh-Ott SL Daniel GB . Histotripsy ablation of spontaneously occurring canine bone tumors. IEEE Trans BioMed Eng. (2023) 70:331–42. doi: 10.1109/TBME.2022.3191069, PMID: 35834467 PMC9921194

[20] Ruger LN Hay AN Vickers ER Coutermarsh-Ott SL Gannon JM Covell HS . Characterizing the ablative effects of histotripsy for osteosarcoma: *in vivo* study in dogs. Cancers. (2023) 15:741. doi: 10.3390/cancers15030741, PMID: 36765700 PMC9913343

[21] Vickers ER Ruger LN Coutermarsh-Ott SL Daniel GB Allen SP Ziemlewicz TJ . MRI for the assessment of histotripsy ablation in a canine osteosarcoma comparative oncology model. Ultrasound Med Biol.(2026) 52:216–26. doi: 10.1016/j.ultrasmedbio.2025.09.017, PMID: 41109828 PMC13375396

[22] Xu Z Hall TL Vlaisavljevich E Lee FT . Histotripsy: the first noninvasive, non-ionizing, non-thermal ablation technique based on ultrasound. Int J Hyperth. (2021) 38:561–75. doi: 10.1080/02656736.2021.1905189, PMID: 33827375 PMC9404673

[23] Bader KB Vlaisavljevich E Maxwell AD . For whom the bubble grows: physical principles of bubble nucleation and dynamics in histotripsy ultrasound therapy. Ultrasound Med Biol. (2019) 45:1056–80. doi: 10.1016/j.ultrasmedbio.2018.10.035, PMID: 30922619 PMC6524960

[24] Vlaisavljevich E Greve J Cheng X Ives K Shi J Jin L . Non-invasive ultrasound liver ablation using histotripsy: chronic study in an *in vivo* rodent model. Ultrasound Med Biol. (2016) 42:1890–902. doi: 10.1016/j.ultrasmedbio.2016.03.018, PMID: 27140521 PMC4912895

[25] Vlaisavljevich E Kim Y Allen S Owens G Pelletier S Cain C . Image-guided non-invasive ultrasound liver ablation using histotripsy: feasibility study in an *in vivo* porcine model. Ultrasound Med Biol. (2013) 39:1398–409. doi: 10.1016/j.ultrasmedbio.2013.02.005, PMID: 23683406 PMC3709011

[26] Vlaisavljevich E Kim Y Owens G Roberts W Cain C Xu Z . Effects of tissue mechanical properties on susceptibility to histotripsy-induced tissue damage. Phys Med Biol. (2013) 59:253. doi: 10.1088/0031-9155/59/2/253, PMID: 24351722 PMC4888779

[27] Styn NR Wheat JC Hall TL Roberts WW . Histotripsy of VX-2 tumor implanted in a renal rabbit model. J Endourol. (2010) 24:1145–50. doi: 10.1089/end.2010.0123, PMID: 20575696

[28] Ikink ME Voogt MJ van den Bosch MAAJ Nijenhuis RJ Keserci B Kim YS . Diffusion-weighted magnetic resonance imaging using different b-value combinations for the evaluation of treatment results after volumetric MR-guided high-intensity focused ultrasound ablation of uterine fibroids. Eur Radiol. (2014) 24:2118–27. doi: 10.1007/s00330-014-3274-y, PMID: 24962829

[29] Achari PF Vickers E Ruger L Vlaisavljevich E Tuohy J Collins CJ . Assessment of histotripsy as a bone-sparing tumor ablation technique in ex vivo osteosarcoma tumor-affected limbs. Front Vet Sci. (2026) 12:1652469. doi: 10.3389/fvets.2025.1652469, PMID: 41585516 PMC12827143

[30] Smolock AR Cristescu MM Vlaisavljevich E Gendron-Fitzpatrick A Green C Cannata J . Robotically assisted sonic therapy as a noninvasive nonthermal ablation modality: proof of concept in a porcine liver model. Radiology. (2018) 287:485–93. doi: 10.1148/radiol.2018171544, PMID: 29381870

[31] Knott EA Swietlik JF Longo KC Watson RF Green CM Abel EJ . Robotically-assisted sonic therapy for renal ablation in a live porcine model: initial preclinical results. J Vasc Interv Radiol JVIR. (2019) 30:1293–302. doi: 10.1016/j.jvir.2019.01.023, PMID: 31130365 PMC6925588

[32] Gannon J Imran KM Hendricks-Wenger A Edwards M Covell H Ruger L . Ultrasound-guided noninvasive pancreas ablation using histotripsy: feasibility study in an *in vivo* porcine model. Int J Hyperthermia. (2023) 40:2247187. doi: 10.1080/02656736.2023.2247187, PMID: 37643768 PMC10839746

[33] Gannon J Paul T Imran KM Edwards M Ziemlewicz T Trusiano B . Non-invasive pancreas ablation using histotripsy: pre-clinical safety study in an *in vivo* porcine model. Ultrasound Med Biol. (2025) 52:62–71. doi: 10.1016/j.ultrasmedbio.2025.07.026, PMID: 41046199 PMC12917764

[34] Imran KM Gannon J Morrison HA Tupik JD Tintera B Nagai-Singer MA . Successful *in situ* targeting of pancreatic tumors in a novel orthotopic porcine model using histotripsy. Ultrasound Med Biol. (2023) 49:2361–70. doi: 10.1016/j.ultrasmedbio.2023.07.013, PMID: 37596154 PMC10529075

[35] Vezza C Ruger L Langman M Vickers E Prada F Sukovich J . First-in-DOg HISTotripsy for intracranial tumors trial: the FIDOHIST study. Technol Cancer Res Treat. (2024) 23:15330338241285158. doi: 10.1177/15330338241285158, PMID: 41930703

[36] Ruger L Yang E Coutermarsh-Ott S Vickers E Gannon J Nightengale M . Histotripsy ablation for the treatment of feline injection site sarcomas: a first-in-cat *in vivo* feasibility study. Int J Hyperth. (2023) 40:2210272. doi: 10.1080/02656736.2023.2210272, PMID: 37196996 PMC10278555

[37] Ruger L Yang E Gannon J Sheppard H Coutermarsh-Ott S Ziemlewicz TJ . Mechanical high-intensity focused ultrasound (Histotripsy) in dogs with spontaneously occurring soft tissue sarcomas. IEEE Trans BioMed Eng. (2023) 70:768–79. doi: 10.1109/TBME.2022.3201709, PMID: 36006886 PMC9969335

[38] Worlikar T Hall T Zhang M Mendiratta-Lala M Green M Cho CS . Insights from *in vivo* preclinical cancer studies with histotripsy. Int J Hyperthermia. (2024) 41:2297650. doi: 10.1080/02656736.2023.2297650, PMID: 38214171 PMC11102041

[39] Worlikar T Mendiratta-Lala M Vlaisavljevich E Hubbard R Shi J Hall TL . Effects of histotripsy on local tumor progression in an *in vivo* orthotopic rodent liver tumor model. BME Front. (2020) 2020:1–14. doi: 10.34133/2020/9830304, PMID: 34327513 PMC8318009

[40] Worlikar T Zhang M Ganguly A Hall TL Shi J Zhao L . Impact of histotripsy on development of intrahepatic metastases in a rodent liver tumor model. Cancers. (2022) 14:1612. doi: 10.3390/cancers14071612, PMID: 35406383 PMC8996987

[41] Pepple AL Guy JL McGinnis R Felsted AE Song B Hubbard R . Spatiotemporal local and abscopal cell death and immune responses to histotripsy focused ultrasound tumor ablation. Front Immunol. (2023) 14:1012799. doi: 10.3389/fimmu.2023.1012799, PMID: 36756111 PMC9900174

[42] Qu S Worlikar T Felsted AE Ganguly A Beems MV Hubbard R . Non-thermal histotripsy tumor ablation promotes abscopal immune responses that enhance cancer immunotherapy. J Immunother Cancer. (2020) 8:e000200. doi: 10.1136/jitc-2019-000200, PMID: 31940590 PMC7057529

[43] Duclos S Golin A Fox A Chaudhary N Camelo-Piragua S Pandey A . Transcranial histotripsy parameter study in primary and metastatic murine brain tumor models. Int J Hyperthermia. (2023) 40:2237218. doi: 10.1080/02656736.2023.2237218, PMID: 37495214 PMC10410615

[44] Choi SW Duclos S Camelo-Piragua S Chaudhary N Sukovich J Hall T . Histotripsy treatment of murine brain and glioma: temporal profile of magnetic resonance imaging and histological characteristics post-treatment. Ultrasound Med Biol. (2023) 49:1882–91. doi: 10.1016/j.ultrasmedbio.2023.05.002, PMID: 37277304 PMC11894760

[45] Iwanicki I Wu LL Flores-Guzman F Sundland R Viza-Gomes P Nordgren R . Histotripsy induces apoptosis and reduces hypoxia in a neuroblastoma xenograft model. Int J Hyperthermia. (2023) 40:2222941. doi: 10.1080/02656736.2023.2222941, PMID: 37344380

[46] Hendricks-Wenger A Howell J Schmieley R Kozlov S Simon A Coutermarsh-Ott S . Histotripsy initiates local and systemic immunological response and reduces tumor burden in breast cancer. J Immunol. (2019) 202:194.30–0. doi: 10.4049/jimmunol.202.Supp.194.30, PMID: 34749531

[47] Hendricks-Wenger A Sereno J Gannon J Zeher A Brock RM Beitel-White N . Histotripsy ablation alters the tumor microenvironment and promotes immune system activation in a subcutaneous model of pancreatic cancer. IEEE Trans Ultrason Ferroelectr Freq Control. (2021) 68:2987–3000. doi: 10.1109/TUFFC.2021.3078094, PMID: 33956631 PMC9295194

[48] Vidal-Jove J Serres X Vlaisavljevich E Cannata J Duryea A Miller R . First-in-man histotripsy of hepatic tumors: the THERESA trial, a feasibility study. Int J Hyperthermia. (2022) 39:1115–23. doi: 10.1080/02656736.2022.2112309, PMID: 36002243

[49] Mendiratta-Lala M Wiggermann P Pech M Serres-Créixams X White SB Davis C . The HOPE4LIVER single-arm pivotal trial for histotripsy of primary and metastatic liver tumors. Radiology. (2024) 312:e233051. doi: 10.1148/radiol.233051, PMID: 39225612 PMC11427859

[50] Hay AN Imran KM Hendricks-Wenger A Gannon JM Sereno J Simon A . Ablative and immunostimulatory effects of histotripsy ablation in a murine osteosarcoma model. Biomedicines. (2023) 11:10. doi: 10.3390/biomedicines11102737, PMID: 37893110 PMC10604356

[51] Hay AN Simon A Ruger LN Gannon J Coutermarsh-Ott S Vickers ER . Establishing human and canine xenograft murine osteosarcoma models for application of focused ultrasound ablation. Biomedicines. (2025) 13:2122. doi: 10.3390/biomedicines13092122, PMID: 41007685 PMC12467533

[52] Stribbling SM Beach C Ryan AJ . Orthotopic and metastatic tumour models in preclinical cancer research. Pharmacol Ther. (2024) 257:108631. doi: 10.1016/j.pharmthera.2024.108631, PMID: 38467308 PMC11781865

[53] Pu F Guo H Shi D Chen F Peng Y Huang X . The generation and use of animal models of osteosarcoma in cancer research. Genes Dis. (2024) 11:664–74. doi: 10.1016/j.gendis.2022.12.021, PMID: 37692517 PMC10491873

[54] Couillard AB Zlevor AM Ziemlewicz TJ Kisting MA Knott E Rossebo AE . A comparison of histotripsy and percutaneous cryoablation in a chronic healthy swine kidney model. J Vasc Interv Radiol. (2023) 34:1986–96. doi: 10.1016/j.jvir.2023.07.014, PMID: 37481064

[55] Sottnik JL Duval DL J Ehrhart E Thamm DH . An orthotopic, postsurgical model of luciferase transfected murine osteosarcoma with spontaneous metastasis. Clin Exp Metastasis. (2010) 27:151–60. doi: 10.1007/s10585-010-9318-z, PMID: 20213324

[56] Jacques C Renema N Lezot F Ory B Walkley CR Grigoriadis AE . Small animal models for the study of bone sarcoma pathogenesis:characteristics, therapeutic interests and limitations. J Bone Oncol. (2018) 12:7–13. doi: 10.1016/j.jbo.2018.02.004, PMID: 29850398 PMC5966525

[57] Maxwell AD Cain CA Hall TL Fowlkes JB Xu Z . Probability of cavitation for single ultrasound pulses applied to tissues and tissue-mimicking materials. Ultrasound Med Biol. (2013) 39:449–65. doi: 10.1016/j.ultrasmedbio.2012.09.004, PMID: 23380152 PMC3570716

[58] Vlaisavljevich E Lin KW Maxwell A Warnez MT Mancia L Singh R . Effects of ultrasound frequency and tissue stiffness on the histotripsy intrinsic threshold for cavitation. Ultrasound Med Biol. (2015) 41:1651–67. doi: 10.1016/j.ultrasmedbio.2015.01.028, PMID: 25766571 PMC4426049

[59] Blattmann C Thiemann M Stenzinger A Roth EK Dittmar A Witt H . Establishment of a patient-derived orthotopic osteosarcoma mouse model. J Transl Med. (2015) 13:136. doi: 10.1186/s12967-015-0497-x, PMID: 25926029 PMC4428092

[60] Vormoor B Knizia HK Batey MA Almeida GS Wilson I Dildey P . Development of a preclinical orthotopic xenograft model of ewing sarcoma and other human Malignant bone disease using advanced *in vivo* imaging. PLoS One. (2014) 9:e85128. doi: 10.1371/journal.pone.0085128, PMID: 24409320 PMC3883696

[61] Zimmer WD Berquist TH McLeod RA Sim FH Pritchard DJ Shives TC . Bone tumors: magnetic resonance imaging versus computed tomography. Radiology. (1985) 155:709–18. doi: 10.1148/radiology.155.3.4001374, PMID: 4001374

[62] Zimmer WD Berquist TH McLeod RA Sim FH Pritchard DJ Shives TC . Magnetic resonance imaging of osteosarcomas. Comparison with computed tomography. Clin Orthop. (1986), 289–99. doi: 10.1097/00003086-198607000-00050, PMID: 3459602

[63] Wallack ST Wisner ER Werner JA Walsh PJ Kent MS Fairley RA . Accuracy of magnetic resonance imaging for estimating intramedullary osteosarcoma extent in pre-operative planning of canine limb-salvage procedures. Vet Radiol Ultrasound. (2002) 43:432–41. doi: 10.1111/j.1740-8261.2002.tb01030.x, PMID: 12375777

[64] Davis GJ Kapatkin AS Craig LE Heins GS Wortman JA . Comparison of radiography, computed tomography, and magnetic resonance imaging for evaluation of appendicular osteosarcoma in dogs. J Am Vet Med Assoc. (2002) 220:1171–6. doi: 10.2460/javma.2002.220.1171, PMID: 11990963

[65] Yildirim O Al Khatalin M Kargin OA Camurdan VB . MRI for evaluation of preoperative chemotherapy in osteosarcoma. Radiogr Lond Engl 1995. (2022) 28:593–604. doi: 10.1016/j.radi.2022.04.008, PMID: 35537246

[66] Halalsheh H Amer S Sultan I . Progression before local control in osteosarcoma: Outcome and prognosis-predictive factors. Pediatr Blood Cancer. (2023) 70:e30649. doi: 10.1002/pbc.30649, PMID: 37638816

