# Supplementary figures and images for: Histotripsy treatment reduces tumor burden and extends survival in an orthotopic mouse model of osteosarcoma
Source: Front Oncol. 2026 Apr 13;16:1807753. doi: 10.3389/fonc.2026.1807753 (PMC13111011; doi:10.3389/fonc.2026.1807753)

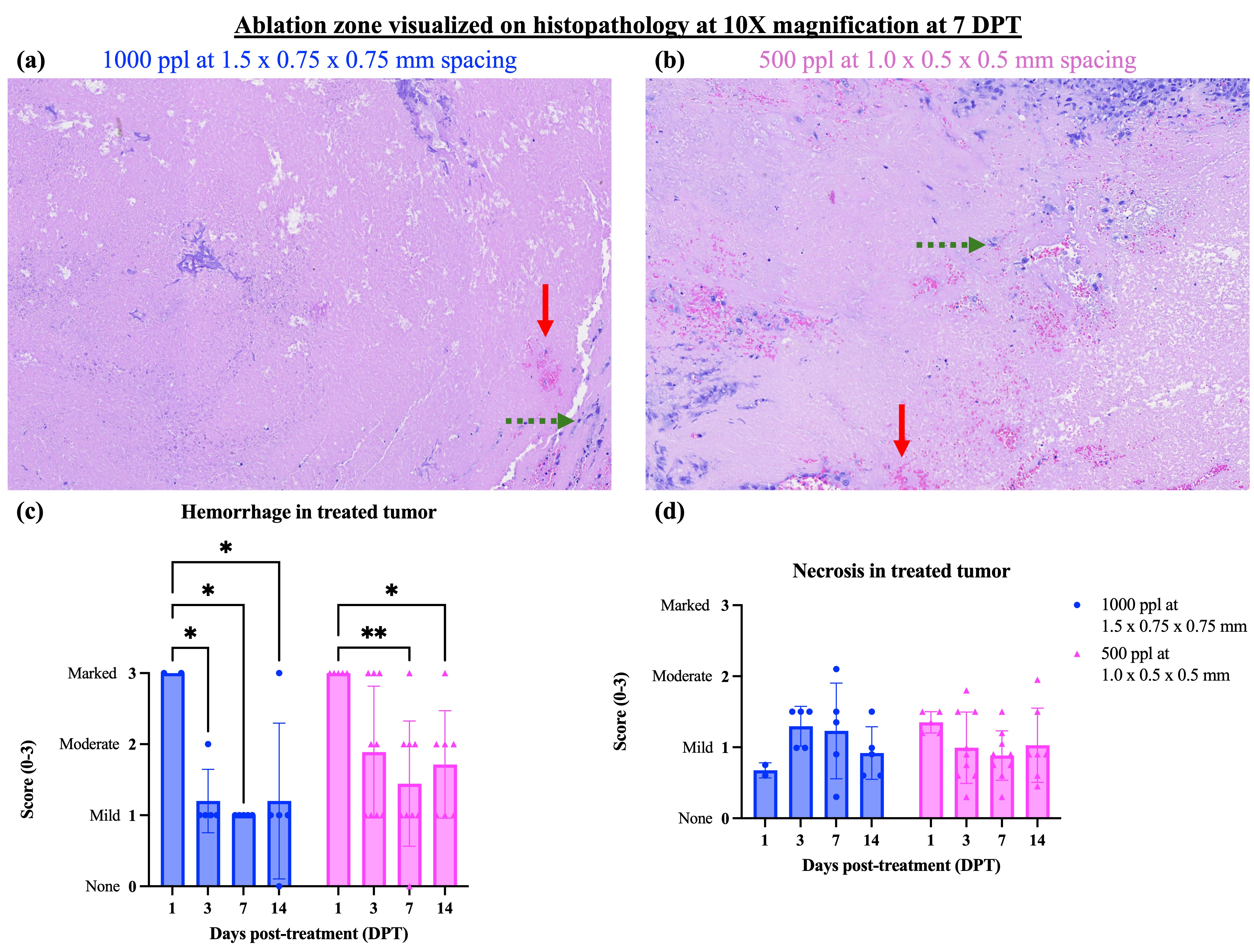

Supplement: Supplementary Figure 1 — Qualitative (a–b) and quantitative (c–d) comparisons between the two different treatment paradigms used in this study. For qualitative data, all histopathology images were routinely stained with H&E; red solid arrows indicate hemorrhage, while green dashed arrows indicate necrotic tumor cells. For quantitative data, two-way ANOVAs with Tukey’s multiple comparisons were used to assess statistical significance for hemorrhage and necrosis as scored on histopathology; * indicates p < 0.05, and ** indicates p < 0.005. Blue circles indicate the 1000 ppl at 1.5 x 0.75 x 0.75 mm spacing, while pink triangles indicate the 500 ppl at 1.0 x 0.5 x 0.5 mm spacing. (a) Representative histopathology at 10X magnification of the ablation zone at 7 DPT for the 1000 ppl at 1.5 x 0.75 x 0.75 mm spacing group. (b) Representative histopathology at 10X magnification of the ablation zone at 7 DPT for the 500 ppl at 1.0 x 0.5 x 0.5 mm spacing group. (c) Hemorrhage as scored on histopathology between the two treatment paradigms. (d) Necrosis as scored on histopathology between the two treatment paradigms. [file DataSheet1.zip › Data Sheet 1/FigureS1.jpg]

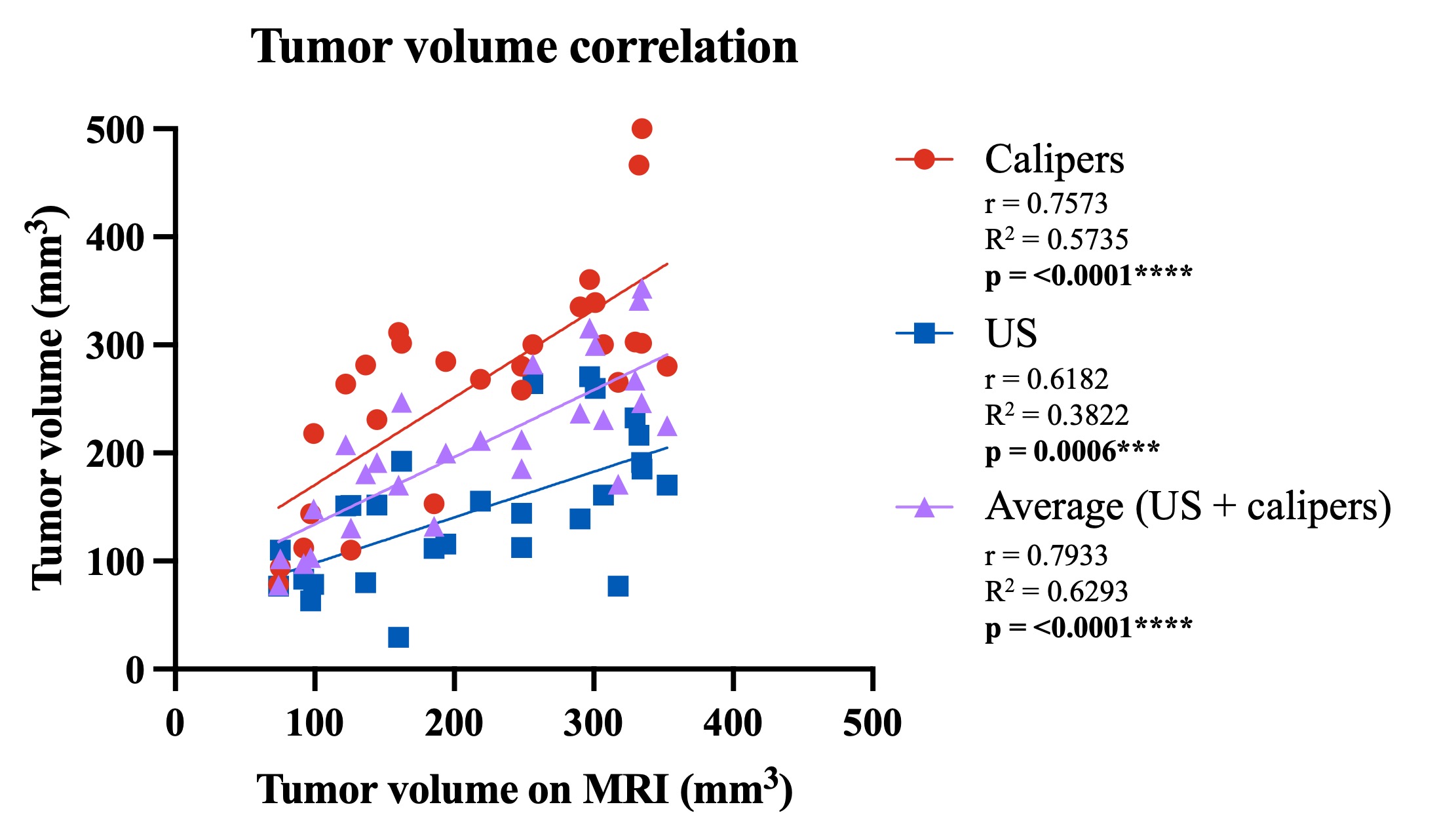

Supplement: Supplementary Figure 1 — Qualitative (a–b) and quantitative (c–d) comparisons between the two different treatment paradigms used in this study. For qualitative data, all histopathology images were routinely stained with H&E; red solid arrows indicate hemorrhage, while green dashed arrows indicate necrotic tumor cells. For quantitative data, two-way ANOVAs with Tukey’s multiple comparisons were used to assess statistical significance for hemorrhage and necrosis as scored on histopathology; * indicates p < 0.05, and ** indicates p < 0.005. Blue circles indicate the 1000 ppl at 1.5 x 0.75 x 0.75 mm spacing, while pink triangles indicate the 500 ppl at 1.0 x 0.5 x 0.5 mm spacing. (a) Representative histopathology at 10X magnification of the ablation zone at 7 DPT for the 1000 ppl at 1.5 x 0.75 x 0.75 mm spacing group. (b) Representative histopathology at 10X magnification of the ablation zone at 7 DPT for the 500 ppl at 1.0 x 0.5 x 0.5 mm spacing group. (c) Hemorrhage as scored on histopathology between the two treatment paradigms. (d) Necrosis as scored on histopathology between the two treatment paradigms. [file DataSheet1.zip › Data Sheet 1/FigureS3.jpg]

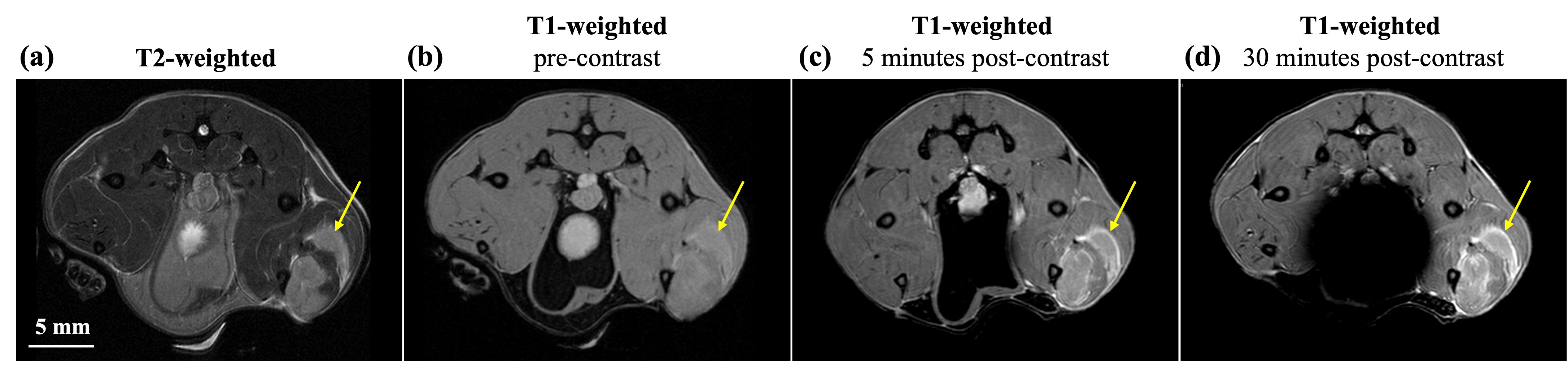

Supplement: Supplementary Figure 1 — Qualitative (a–b) and quantitative (c–d) comparisons between the two different treatment paradigms used in this study. For qualitative data, all histopathology images were routinely stained with H&E; red solid arrows indicate hemorrhage, while green dashed arrows indicate necrotic tumor cells. For quantitative data, two-way ANOVAs with Tukey’s multiple comparisons were used to assess statistical significance for hemorrhage and necrosis as scored on histopathology; * indicates p < 0.05, and ** indicates p < 0.005. Blue circles indicate the 1000 ppl at 1.5 x 0.75 x 0.75 mm spacing, while pink triangles indicate the 500 ppl at 1.0 x 0.5 x 0.5 mm spacing. (a) Representative histopathology at 10X magnification of the ablation zone at 7 DPT for the 1000 ppl at 1.5 x 0.75 x 0.75 mm spacing group. (b) Representative histopathology at 10X magnification of the ablation zone at 7 DPT for the 500 ppl at 1.0 x 0.5 x 0.5 mm spacing group. (c) Hemorrhage as scored on histopathology between the two treatment paradigms. (d) Necrosis as scored on histopathology between the two treatment paradigms. [file DataSheet1.zip › Data Sheet 1/FigureS2.jpg]

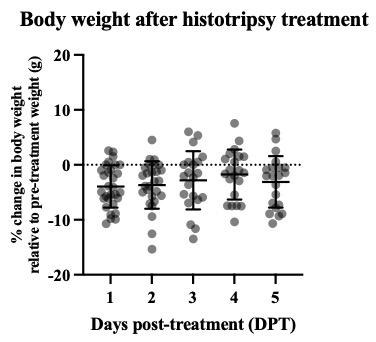

Supplement: Supplementary Figure 1 — Qualitative (a–b) and quantitative (c–d) comparisons between the two different treatment paradigms used in this study. For qualitative data, all histopathology images were routinely stained with H&E; red solid arrows indicate hemorrhage, while green dashed arrows indicate necrotic tumor cells. For quantitative data, two-way ANOVAs with Tukey’s multiple comparisons were used to assess statistical significance for hemorrhage and necrosis as scored on histopathology; * indicates p < 0.05, and ** indicates p < 0.005. Blue circles indicate the 1000 ppl at 1.5 x 0.75 x 0.75 mm spacing, while pink triangles indicate the 500 ppl at 1.0 x 0.5 x 0.5 mm spacing. (a) Representative histopathology at 10X magnification of the ablation zone at 7 DPT for the 1000 ppl at 1.5 x 0.75 x 0.75 mm spacing group. (b) Representative histopathology at 10X magnification of the ablation zone at 7 DPT for the 500 ppl at 1.0 x 0.5 x 0.5 mm spacing group. (c) Hemorrhage as scored on histopathology between the two treatment paradigms. (d) Necrosis as scored on histopathology between the two treatment paradigms. [file DataSheet1.zip › Data Sheet 1/FigureS6.jpg]

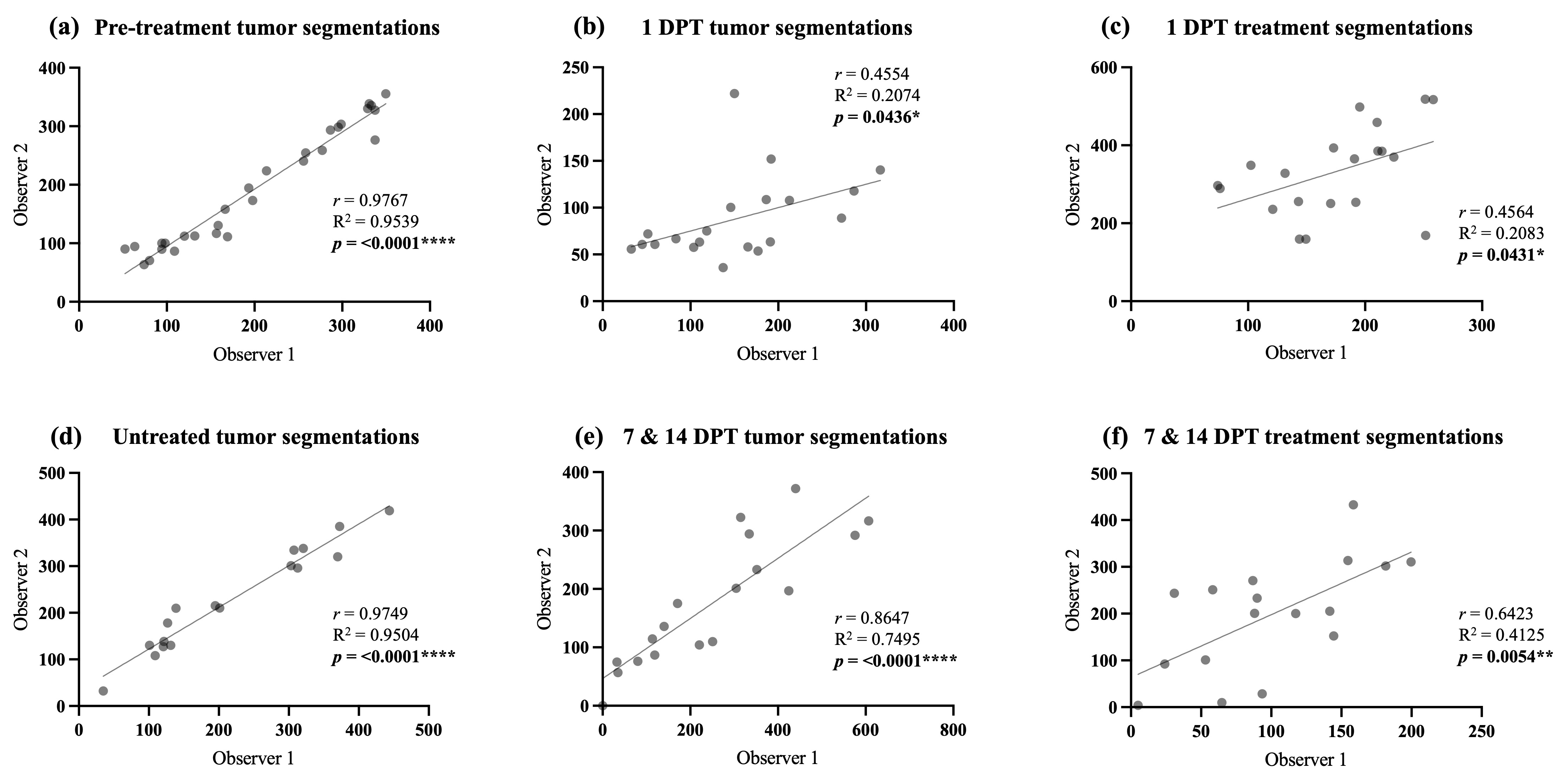

Supplement: Supplementary Figure 1 — Qualitative (a–b) and quantitative (c–d) comparisons between the two different treatment paradigms used in this study. For qualitative data, all histopathology images were routinely stained with H&E; red solid arrows indicate hemorrhage, while green dashed arrows indicate necrotic tumor cells. For quantitative data, two-way ANOVAs with Tukey’s multiple comparisons were used to assess statistical significance for hemorrhage and necrosis as scored on histopathology; * indicates p < 0.05, and ** indicates p < 0.005. Blue circles indicate the 1000 ppl at 1.5 x 0.75 x 0.75 mm spacing, while pink triangles indicate the 500 ppl at 1.0 x 0.5 x 0.5 mm spacing. (a) Representative histopathology at 10X magnification of the ablation zone at 7 DPT for the 1000 ppl at 1.5 x 0.75 x 0.75 mm spacing group. (b) Representative histopathology at 10X magnification of the ablation zone at 7 DPT for the 500 ppl at 1.0 x 0.5 x 0.5 mm spacing group. (c) Hemorrhage as scored on histopathology between the two treatment paradigms. (d) Necrosis as scored on histopathology between the two treatment paradigms. [file DataSheet1.zip › Data Sheet 1/FigureS7.jpg]

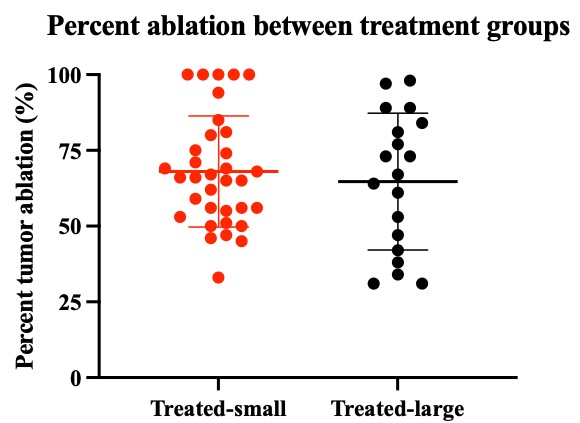

Supplement: Supplementary Figure 1 — Qualitative (a–b) and quantitative (c–d) comparisons between the two different treatment paradigms used in this study. For qualitative data, all histopathology images were routinely stained with H&E; red solid arrows indicate hemorrhage, while green dashed arrows indicate necrotic tumor cells. For quantitative data, two-way ANOVAs with Tukey’s multiple comparisons were used to assess statistical significance for hemorrhage and necrosis as scored on histopathology; * indicates p < 0.05, and ** indicates p < 0.005. Blue circles indicate the 1000 ppl at 1.5 x 0.75 x 0.75 mm spacing, while pink triangles indicate the 500 ppl at 1.0 x 0.5 x 0.5 mm spacing. (a) Representative histopathology at 10X magnification of the ablation zone at 7 DPT for the 1000 ppl at 1.5 x 0.75 x 0.75 mm spacing group. (b) Representative histopathology at 10X magnification of the ablation zone at 7 DPT for the 500 ppl at 1.0 x 0.5 x 0.5 mm spacing group. (c) Hemorrhage as scored on histopathology between the two treatment paradigms. (d) Necrosis as scored on histopathology between the two treatment paradigms. [file DataSheet1.zip › Data Sheet 1/FigureS4.jpg]
